# Composite Photocatalysts Containing BiVO_4_ for Degradation of Cationic Dyes

**DOI:** 10.1038/s41598-017-09514-5

**Published:** 2017-08-21

**Authors:** Kanlaya Pingmuang, Jun Chen, Wiyong Kangwansupamonkon, Gordon G. Wallace, Sukon Phanichphant, Andrew Nattestad

**Affiliations:** 1 ARC Centre of Excellence for Electromaterials Science, Intelligent Polymer Research Institute, Australian Institute of Innovative materials, Innovation Campus, University of Wollongong, Fairy Meadow, NSW 2519 Australia; 2 Department of Chemistry and Materials Science Research Center, Faculty of Science, Chiang Mai University, 239 Huay Kaew Road, Muang District, Chiang Mai, 50200 Thailand; 3 National Nanotechnology Center, Thailand, 130 Thailand Science Park, Paholyothin Road, Pathumthani, 12120 Thailand

## Abstract

The creation of composite structures is a commonly employed approach towards enhanced photocatalytic performance, with one of the key rationales for doing this being to separate photoexcited charges, affording them longer lifetimes in which to react with adsorbed species. Here we examine three composite photocatalysts using either WO_3_, TiO_2_ or CeO_2_ with BiVO_4_ for the degradation of model dyes Methylene Blue and Rhodamine B. Each of these materials (WO_3_, TiO_2_ or CeO_2_) has a different band edge energy offset with respect to BiVO_4_, allowing for a systematic comparison of these different arrangements. It is seen that while these offsets *can* afford beneficial charge transfer (CT) processes, they can also result in the deactivation of certain reactions. We also observed the importance of localized dye concentrations, resulting from a strong affinity between it and the surface, in attaining high overall photocatalytic performance, a factor not often acknowledged. It is hoped in the future that these observations will assist in the judicious selection of semiconductors for use as composite photocatalysts.

## Introduction

Bismuth vanadate (BiVO_4_) has attracted much attention as a highly responsive, visible-light driven, photocatalyst due to its comparatively narrow band gap (E_G_) energy of 2.4 eV, (as compared to TiO_2_, which remains a benchmark, with an E_G_ of 3.0–3.2 eV). BiVO_4_ has been widely used in the photocatalytic degradation of organic compounds in waste water as well as for O_2_ evolution under sunlight irradiation^1–4^.

One of limitations of the photocatalytic efficiency in BiVO_4_ is the recombination of photogenerated electrons and holes, with the free carrier life time reported to be about 40 ns by Abdi *et al*.^5^. In order to enhance these lifetimes, the creation of electronic barriers, facilitating spatial separation of the photogenerated electrons and holes, has been embraced by researchers as they can retard this recombination, providing more opportunities for free holes and electrons to participate in reduction and/or oxidation reactions, such as for the degradation of organic materials^4, 6^. Doping semiconductors, either with metal or non-metals, has also been demonstrated to be an effective method for enhancing photocatalytic performance^4, 6, 7^. The composite approach relies on exploiting band-edge offsets, directing the electrons and holes into different materials, thereby providing spatial separation.

Recently, many publications have coupled BiVO_4_ with other metal oxides such as Bi_2_O_3_^8, 9^, V_2_O_5_^10, 11^, TiO_2_^12, 13^, WO_3_^11, 14–16^, CdS^17^, CuCr_2_O_4_^18^ and CuWO_4_^19^, for water purification and water spitting applications. The results show that the semiconductor composite photocatalysts were more active than individual catalysts for all photocatalytic degradation of organic pollutants - notwithstanding the fact that it is less likely that composites providing lower photocatalytic performances would be published. While most of these reports on composite catalysts acknowledge the role of band edge offsets, detailed investigations of reaction mechanisms are often not undertaken, nor has the nature of these energy offsets been thoroughly scrutinized^8, 10, 13, 18, 20^.

**Figure 1:**
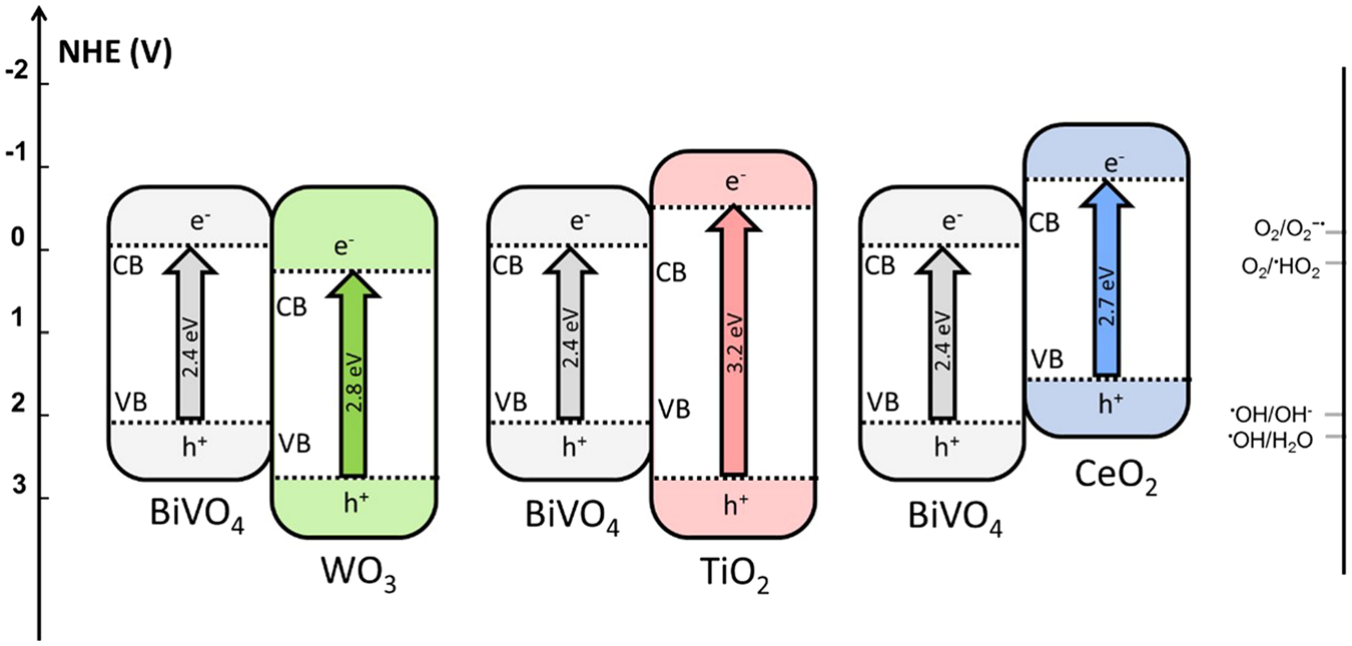
Schematic diagrams of the energy band structures of coupling WO_3_/BiVO_4_, TiO_2_/BiVO_4_ and CeO_2_/BiVO_4_ composites, based on literature values (band edge energy values taken from^6, 7, 20–22^).

Here we present a systematic study, pairing BiVO_4_ with three different metal oxide semiconductors to create different offsets scenarios. CeO_2_/BiVO_4_, TiO_2_/BiVO_4_ and WO_3_/BiVO_4_ provide these different valence band (VB) and conduction band (CB) edge offsets, with the CB and VB potential edges of BiVO_4_ being more positive than those of CeO_2_, in between those of TiO_2_, and more negative than those of WO_3_; as shown in Fig. 1 ^6, 7, 20–22^. These materials are selected based on literature values, as it is expected that this will result in different charge transfer (CT) processes being able to take place. In each case BiVO_4_ has the narrowest bandgap and hence the broadest absorption, so the total amount of light harvested is consistent for each of the composites. As the above band edge potentials are obtained from different sources, and measured under different conditions, optical band gap and Mott-Shotkey measurements are conducted in order to confirm the predicted offsets.

**Figure 2:**
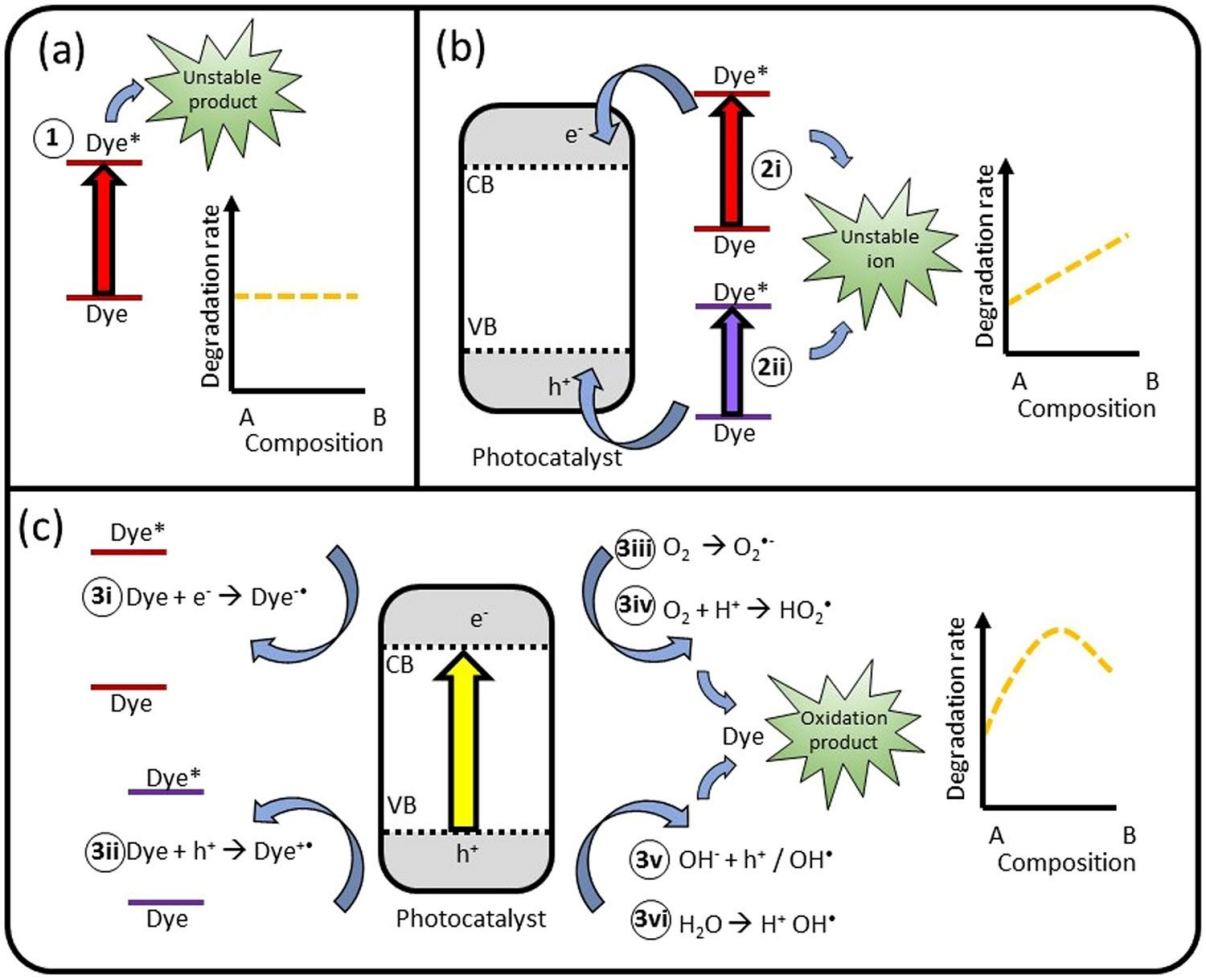
Photodegradation mechanisms. (**a**) Direct degradation by (1) direct photolysis. (**b**) Dye-sensitization leading to degradation through either electron (2i) or hole injection (2ii). (**c**) Indirect degradation of dyes by various photocatalytic reactions. (3i) and (3ii) involve direct oxidation/reduction of the dye by the photoexcited catalyst, while (3ii–3vi) proceed via radical intermediates. For each class of catalytic mechanisms, a schematic (yellow dotted line) represents how they are expected to vary with composition, with further explanation provided in text.

A number of mechanisms for photocatalytic degradation of dyes by semiconductors are summarised in Fig. 2, including (1) direct photolysis of dyes (2) dye photosensitization (*i.e*. charge injection from a photoexcited dye leaving unstable dye anion/cation) and (3) photocatalytic degradation of dyes attack by radical generated by photoexcitation of semiconductor (SC)^22–25^. Typically direct photolysis is very slow, impractical as a means to remove organic contaminants from waste water, and indeed is the reason why researchers look towards using photocatalysts to enhance degradation rates^24^. Figure 2a shows schematically how mechanism (1) operates. It is expected that mechanism (1) should be independent of the catalyst used, or the percentage of each catalysts used in composite systems, provided there is no light harvesting competition (Methylene Blue, MB, absorbs most strongly at 664 nm, well beyond the absorption onset of BiVO_4_).

There are two variants of mechanism (2) which may occur, depending on the redox potentials of the dye compared to the band edges of the catalyst. Mechanism (2i) is photoanodic sensitization, with the excited dye injecting an electron into the semiconductor leading to an oxidized radial cation (Dye^+•^), while (2ii) is photocathodic sensitization, generating a reduced radical dye (Dye^−•^). Furthermore, these injected charges may lead to generation of other radical species. The chemical driving force between the dye redox potentials and those of the semiconductor will largely determine the rate of mechanism (2), and as such, with everything else being equal, should be proportional to the geometric mean of the components in composites.

Degradation mechanisms (3i-vi), shown in Fig. 2b, rely on photoexcitation of the semiconductor. Generated h^+^ and e^−^ pairs (in the VB and CB of the semiconductor respectively) then react either directly (3i and 3ii) or generate radical intermediate species, which can degrade organic species such as the aforementioned dyes (3iii–3vi). There are a number of reactions which may take place, once again depending on the energies of the semiconductor band edges and the environment in which it exists^22, 26–28^:

(3i)e^−^_CB_ + Dye → Dye^−•^ (Dye dependent)

(3ii)h^+^_VB_ + Dye → Dye^+•^ (Dye dependent)

(3iii)e^−^_CB_ + O_2(aq)_ → O_2_^−•^ (−0.18 V *vs*. NHE)

(3iv)e^−^_CB_ + O_2(aq)_ + H^+^ → HO_2_^•^ (+0.10 V *vs*. NHE)

(3v)h^+^_VB_ + OH^−^ → OH^•^ (+1.99 V *vs*. NHE)

(3vi)h^+^_VB_ + H_2_O → H^+^  + OH^•^ (+2.73 V *vs*. NHE)

Thus, the degradation will greatly depend on the adsorption of the dye, O_2_, OH^−^, H^+^ and/or H_2_O on the surface of semiconductor. Photogenerated e^−^ and h^+^ can of course also recombine (either radiatively or non-radiatively) within the material. It is therefore beneficial to increase the lifetimes of these species to increase the probability that they will participate in one of the above reactions (3i-vi). Mechanisms in this third family may display a synergistic response, where composites can lead to higher reaction rates than either of the two component materials.

In addition to examining rates of photocatalytic degradation for the different heterostructures, in this study, we also examine the mechanisms. This is done in two ways; firstly, through reactive species quenching studies, to differentiate between mechanisms involving OH^•^, O_2_^•−^, h^+^, e^−^ and secondly with action spectra, taken to deconvolute the responses of the constituent materials. Additionally, a range of physical characterization techniques were employed, such as X-Ray Diffraction (XRD), X-ray photoelectron spectroscopy (XPS), Mott-Schottky experiments, and Scanning Electron Microscopy (SEM).

Rhodamine B (RhB) and Methylene Blue (MB) are commonly used as model dyes for photodegradation experiments, being broadly representative of organic compounds in their class^22, 24^, while being strongly light absorbing (making the remaining concentration easy to monitor by UV-Vis spectroscopy). In addition, since they absorb visible light, mechanisms (1) - direct photolysis^25^, and (2) - sensitization may occur in these systems, in addition to (3). Photoexcited MB can undergo a one electron reduction by other MB molecules to produce *Leuco*-methylene blue (MB/MB^•−^ = −0.23 V *vs*. NHE)^23^.

## Results

WO_3_/BiVO_4_, TiO_2_/BiVO_4_ and CeO_2_/BiVO_4_ composite powders, along with the respective pure materials, were synthesized by wet chemical methods with different mole ratios (1:4, 2:3, 1:1, 3:2 and 4:1), and used to produce photocatalyst films on glass by a doctor blading method^29^. In a preliminary study, the photocatalytic activities these investigated by degradation of MB and RhB under simulated solar irradiation. The concentration of remaining of dye shows a first order degradation relationship with respect to time, which can be fitted to the Langmuir-Hinshelwood (LH) kinetic model^30^ (shown in Fig. S1). It was found that 1:4, 1:1 and 2:3 were the optimal ratios of WO_3_:BiVO_4_, TiO_2_:BiVO_4_ and CeO_2_:BiVO_4_ respectively for both MB and RhB under visible light irradiation, (Fig. S2). It should be noted that in each case, the composites outperformed the individual component materials, as predicted for type (3) mechanisms. These optimal ratios were chosen for further investigations, and are referred to simply as WO_3_/BiVO_4_, TiO_2_/BiVO_4_ and CeO_2_/BiVO_4_, respectively from this point on.

**Figure 3:**
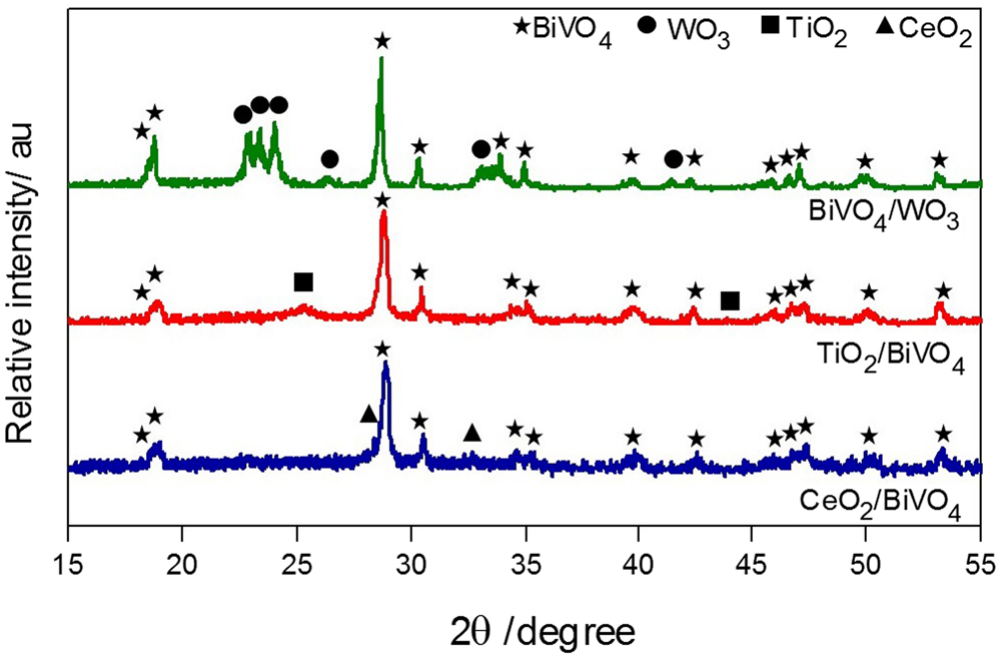
XRD patterns of composite photocatalysts.

The crystal structures of the composites were characterized by XRD, as shown in Fig. 3, with each XRD pattern showing only characteristic diffraction peaks of monoclinic BiVO_4_ and either monoclinic WO_3_, tetragonal TiO_2_ (anatase) or cubic fluorite CeO_2_ respectively, as expected. XRD patterns of the individual component materials can also be found in Fig. S3. Furthermore, XPS analysis was carried out to study the surface chemical composition of the above composites, and take a more detailed look at the interactions between BiVO_4_ and the other metal oxide in each of the composites, as shown in Figs S4–S6. Due to proximity, electronic interactions were observed for these composites when examined by XPS^30–34^. It was therefore concluded, from XRD and XPS that these systems were intimately formed composites, but did not contain any significant doping or any a mixed oxide phases.

**Figure 4:**
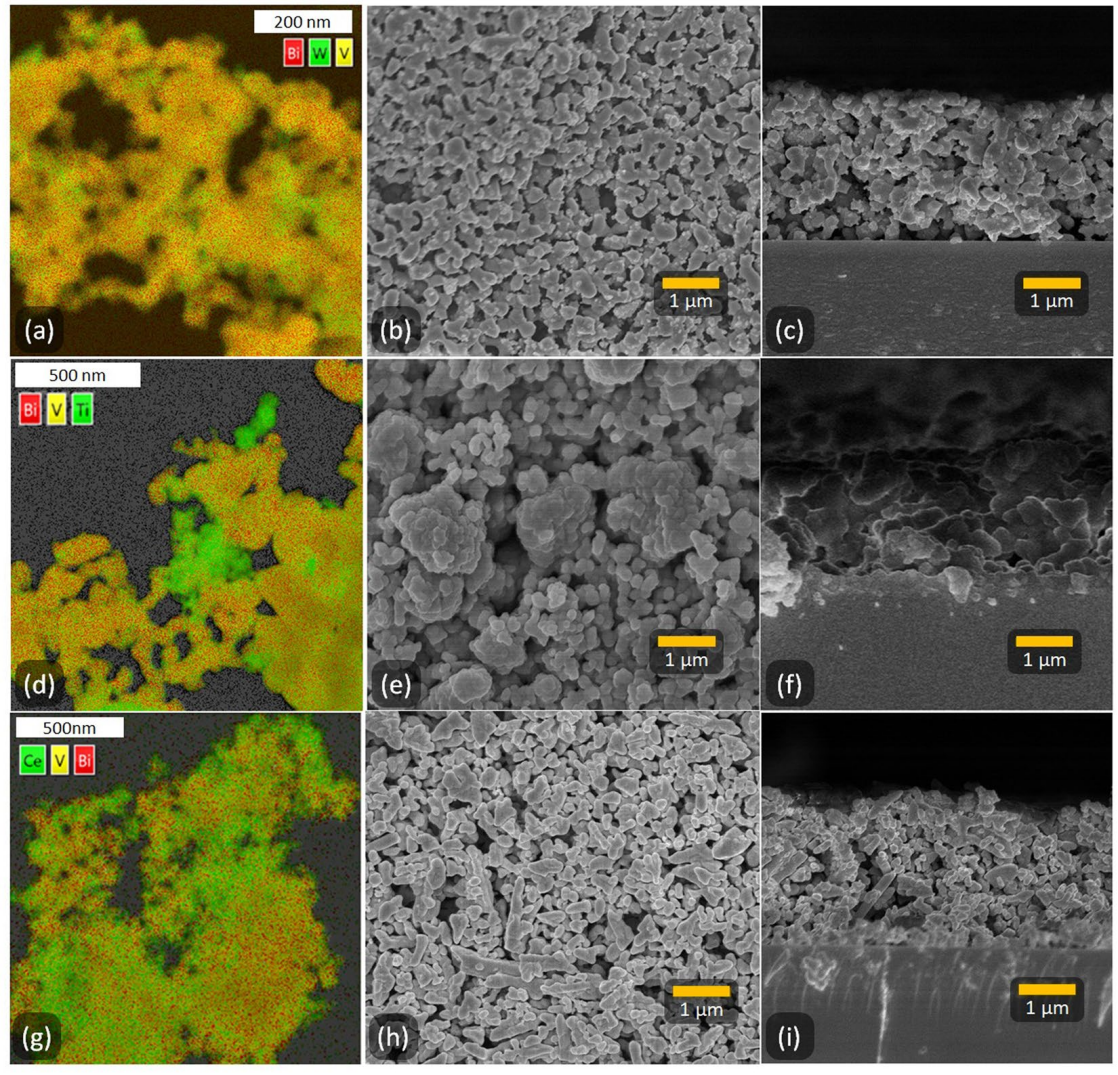
EDS maps of nanocomposite powders (**a**,**d**,**g**), along with top down (**b**,**e**,**h**) and cross-sectional (**c**,**f**,**i**) SEM images of the composite films of (**a**–**c**), WO_3_/BiVO_4_, (**d**–**f**) TiO_2_/BiVO_4_, and (**g**–**i**), CeO_2_/BiVO_4_, respectively.

The morphologies of WO_3_/BiVO_4_, TiO_2_/BiVO_4_ and CeO_2_/BiVO_4_ composite powders and films were investigated using SEM (Fig. 4), with films shown to be highly porous and approximately the same thickness, ~2.8 μm, by cross-sectional SEM (Fig. 4c,f,i), in line with profilometry values mentioned previously. In each case, the two component materials appear to be well distributed among each other, as it particularly evident in the Energy Dispersive X-Ray Spectroscopy (EDS) maps. EDS data shows Bi and V tracking with one another, as expected, while other regions are rich in either W, Ti or Ce respectively, corresponding to WO_3_, TiO_2_ or CeO_2_ particles in the relevant composites. The scale of these differences suggests again that there are well-formed composites, with each containing image (Fig. 4a,d,g) two distinct materials in close proximity to one another. Further analysis of EDS (Figs S7–S9) is included in the supplemental information, along with TEM images (Fig. S10).

**Figure 5:**
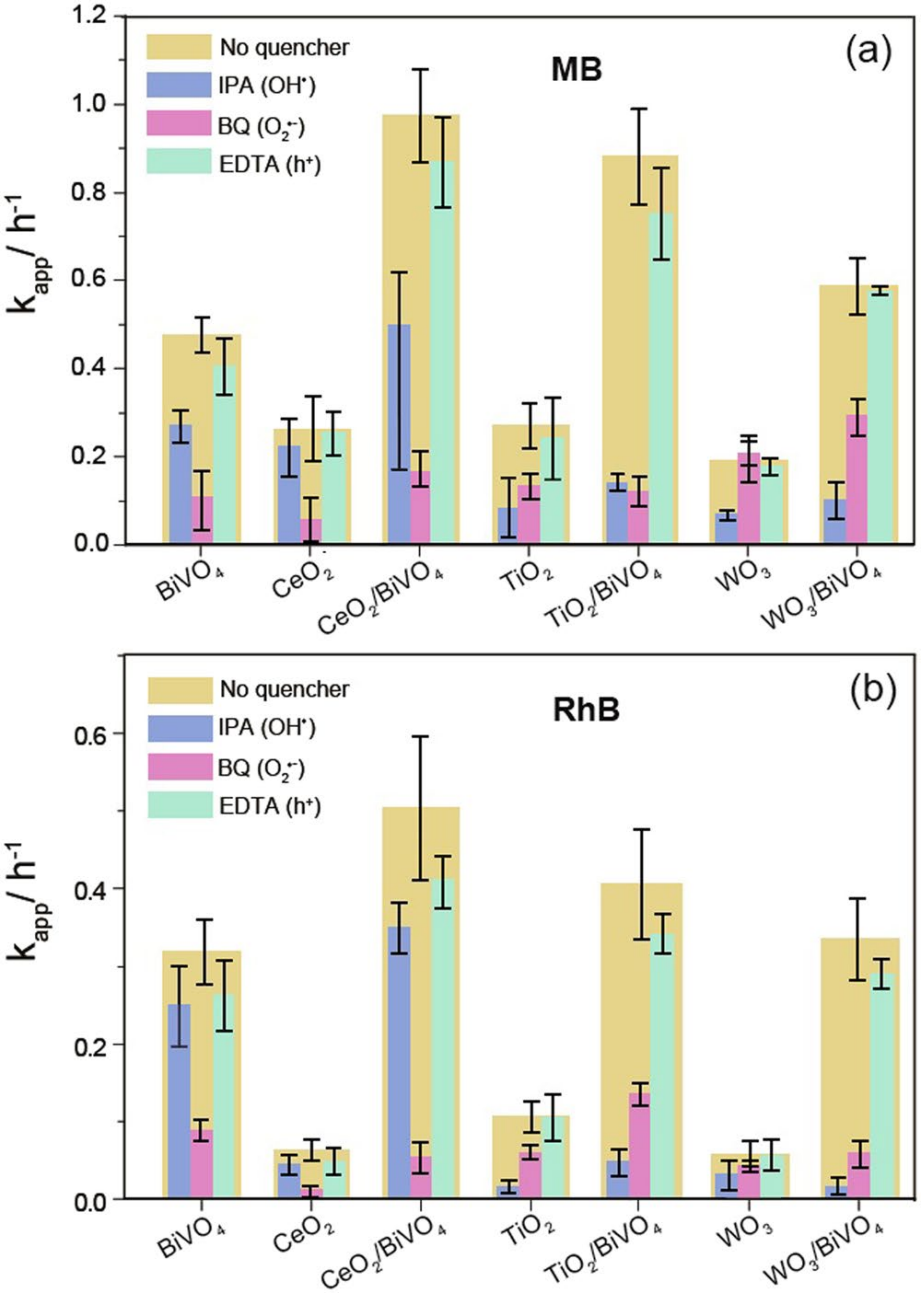
Observed pseudo first order rate constants (k_app_) of photodegradation of (**a**) MB and (**b**) RhB by using different photocatalysts with and without scavengers under simulated solar irradiation.

The mechanisms of photocatalytic degradation of MB and RhB, by BiVO_4_/WO_3_ BiVO_4_/TiO_2_ and BiVO_4_/CeO_2_ were investigated by an indirect chemical probe method, with active species scavengers (see Fig. 5). Normally in the photocatalytic oxidation process, organic species (notably double bonds) are attacked by active species, including holes (h^+^), hydroxyl radical (HO^•^) and/or superoxide anion radical (O_2_^•−^)^35–40^. As mentioned, IPA, BQ and EDTA were introduced into photocatalysis experiment as scavengers of HO^•^, O_2_^•−^ and h^+^, respectively. The photocatalytic degradation rate constant (k_app_) for the composites and pure materials (both for MB (Fig. 5a) and RhB (Fig. 5b)), with the above quenchers (1 mM) were investigated under simulated solar irradiation.

The addition of BQ almost completely quenched the dye degradation of both the pure BiVO_4_ and CeO_2_ indicating that O_2_^•−^ is the major active species in these systems. For the photodegradation of dyes with the TiO_2_ and WO_3_, a large suppression was noted upon the addition of IPA. As such, HO^•^ was shown to be the main oxidation specie in pure TiO_2_ and WO_3_ photocatalysis systems. The photocatalytic systems with added EDTA showed similar degradation rates compared to the system without scavengers, suggesting a limited role played by holes in the valence band. This may, in part, due to these reactions proceeding via reactions with OH^−^, which is presumed to be in short supply at pH 5.

**Figure 6:**
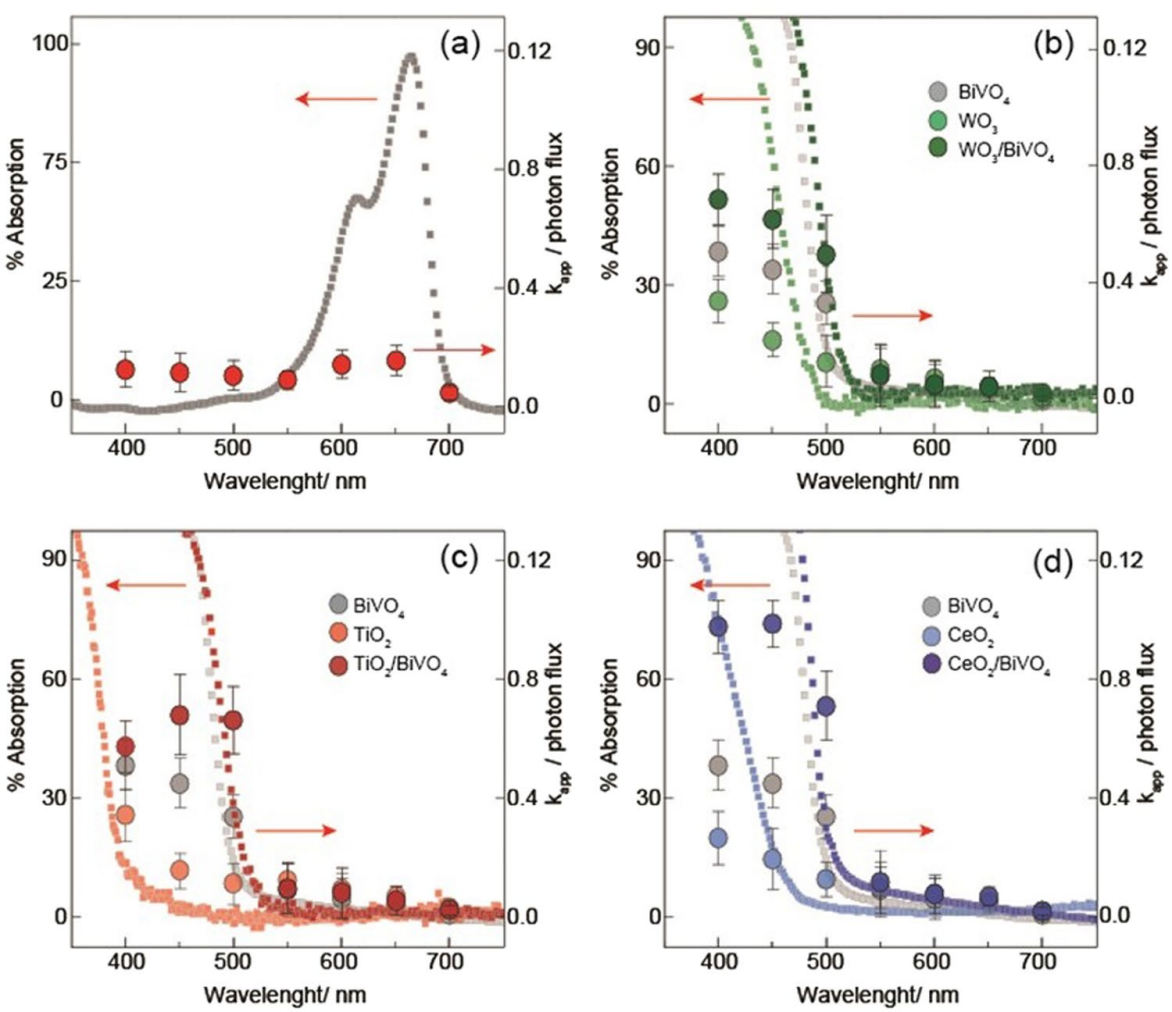
Absorption spectra of photocatalysts, and photocatalytic degradation rate of MB (k_app_) as a function of wavelength (±20 nm band pass filters used), normalised against photon flux; (**a**) no catalyst; (**b**) WO_3_, WO_3_/BiVO_4_ and BiVO_4_; (**c**) TiO_2_, TiO_2_/BiVO_4_ and BiVO_4_; and (**d**) CeO_2_, CeO_2_/BiVO_4_ and BiVO_4_.

Additionally, these studies suggest multiple mechanisms are at play in the degradation of MB and RhB dyes, with further studies being done to confirm this. Two major active species’ in the photodegradation process of MB and RhB dyes in the presence of TiO_2_/BiVO_4_ composite were seen, O_2_^•−^ and HO^•^, while the main species of CeO_2_/BiVO_4_ and BiVO_4_/WO_3_ composite systems were O_2_^•−^ and HO^•^, respectively. The generation of these radical species is explained in the context of their band edge energies, with the conduction band edge energies confirmed by Mott-Schottky measurements (Fig. S11) and valence band edges inferred based on optical bandgaps (see Fig. 6, Fig. S12 and Table S2). These experiments also highlight the possibility of a photocatalyst system being poisoned, a challenge which must be addressed in any real-world application of this technology.

Figure 6 shows the normalised rate constants for photodegradation of MB dye over the different photocatalysts under monochromatic light (using band pass filters) along with their absorption spectra. MB was chosen for this study as its absorption has little overlap with that of the photocatalysts in question, as opposed to RhB. As expected, in the absence of any catalyst (Fig. 6a) MB degraded most rapidly under 650 ± 20 nm light, close to its maximum absorption (664 nm) as a result of direct photolysis (mechanism 1, from Fig. 2a)^22, 41, 42^. A similar response to these wavelengths were seen with all catalysts as well. As was mentioned previously, mechanism (1) degradation should be largely independent of the catalyst used, while mechanism (2, specifically 2i in this case) will be influenced by the chemical potential of free electrons in the CB. It is therefore concluded that that mechanism (2) plays a minor role, if at all, in the total photodegradation process here.

The absorption onsets of the WO_3_, TiO_2_ and CeO_2_ were found to be 500, 390 nm and 435 nm respectively, while the absorption onset of all composites were dictated by that of BiVO_4_, at about 525 nm (shown in Fig. 6b–d). It is seen that the main mechanism of MB degradation in each case is due to the photogeneration of reactive radical species. As mentioned above, composite formation is shown to have synergistic effect on degradation rates, suggesting effective interactions between the various metal oxide species.

For each composite, the responses to light of higher than band gap energies were enhanced as compared to the individual components, however this was more dramatic for some cases than others. As mentioned previously, BiVO_4_ was selected as the common material as this results in equivalent total light harvesting for the composites. This hypothesis was validated looking at the absorption onsets of the composites, which were all indeed close to that of pure BiVO_4_. This also provides us with the ability to deconvolute the responses of the coupled materials. At 500 nm BiVO_4_ absorbs, whereas CeO_2_ or TiO_2_ do not (this is within the range of the absorption onset for WO_3_, however direct comparison the response to this 500 nm light, as compared to 400 nm or 450 nm, where WO_3_ more strongly absorbs light, is illustrative). Interestingly, while all composites showed a synergistic response at this wavelength, in the case of TiO_2_ and WO_3_, shorter wavelengths (400 and 450 nm) result in less marked differences as compared to pure BiVO_4_. On the other hand, CeO_2_ displays similar synergistic behaviour at all wavelengths from 400 to 500 nm.

**Figure 7:**
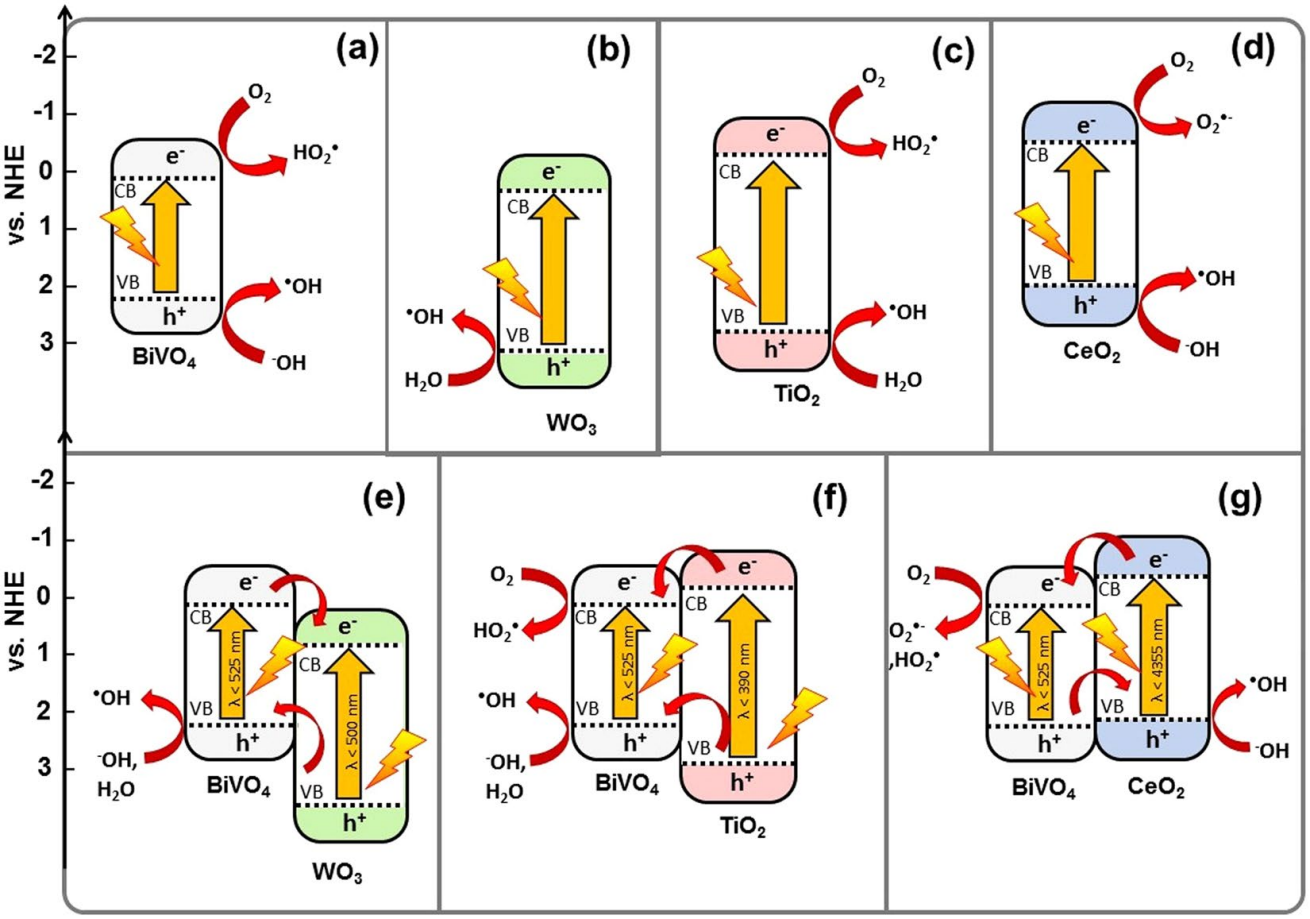
The proposed photocatalytic dye degradation mechanism in the presence of (**a**) BiVO_4_, (**b**) WO_3_, (**c**) TiO_2_, (**d**) CeO_2_, (**e**) WO_3_/BiVO_4_, (**f**) TiO_2_/BiVO_4_, and (**g**) CeO_2_/BiVO_4_ composites, irradiated under simulated solar light.

Based on the combined results of quenching studies, monochromatic illumination Mott-Schottky experiments (Fig. S11) and optical band gap measurements (Fig. S12), the primary degradation mechanisms are proposed for the individual materials and WO_3_/BiVO_4_, TiO_2_/BiVO_4_, and CeO_2_/BiVO_4_ composites and are shown in Fig. 7. As mentioned, the main photodegradation mechanism seen with BiVO_4_ involves the transfer of a photoexcited e^−^ from the CB of BiVO_4_, along with a proton, to adsorbed an O_2_ on the semiconductor surface, generating HO_2_^•^.

Meanwhile, quenching studies showed OH^•^ to the only major reaction species for the pure WO_3_ photocatalyst system, which be produced by oxidation at VB of WO_3_. As OH^−^ exists only at low concentrations in slightly acid conditions, the photocatalytic activity exhibited here was lower than other materials. For the WO_3_/BiVO_4_ composite, (Fig. 7e) the CB and VB band edges of BiVO_4_ are more negative than the CB and VB of the other component. Both BiVO_4_ and WO_3_ can be activated by visible light irradiation and generated e^−^ and h^+^ pairs, although at 500 ± 20 nm, the relative portion of light harvested by the WO_3_ should be considerably less compared to BiVO_4_ than under 400 or 450 nm illumination.

**Figure 8:**
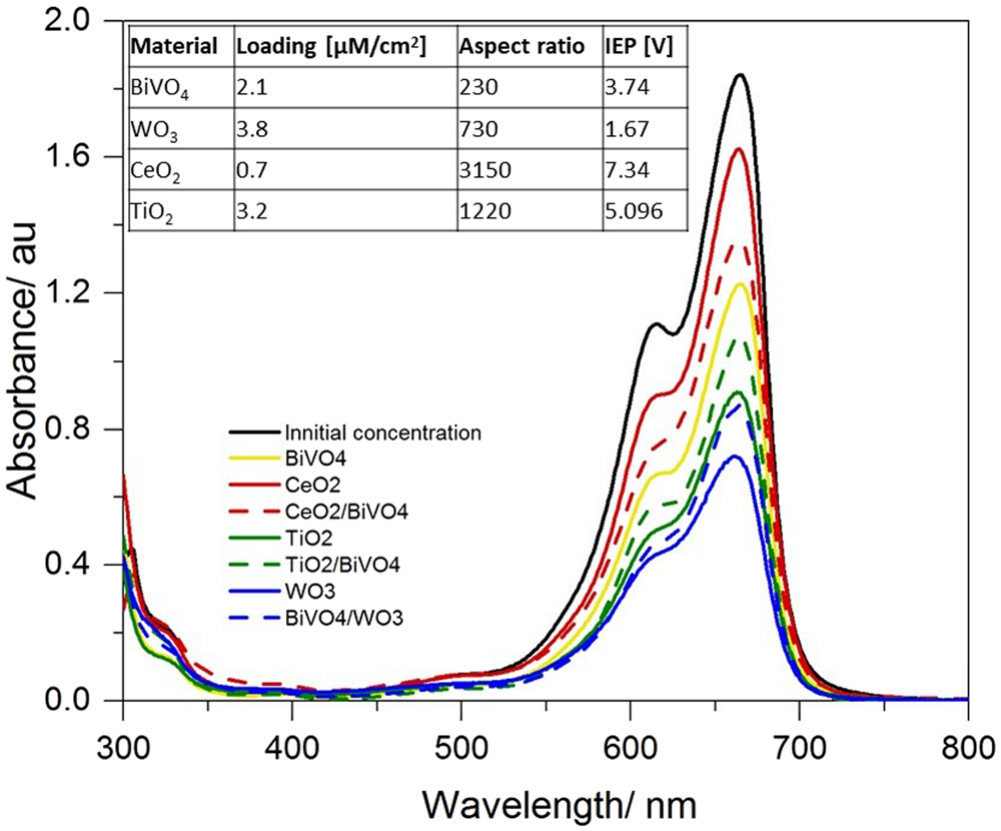
The absorption of MB solutions after being allowed to reach an adsorption-desorption equilibrium with the various photocatalyst films, overnight and in the dark. Inset compares the calculated dye loadings on the materials with their aspect ratios and isoelectric points (IEP).

According to the band edge analysis, electrons transferred to the CB of WO_3_ cannot reduce O_2_ to form radical species, as they can from the BiVO_4_ CB. This is reflected in quenching studies, with the deactivation of the O_2_^−•^ species mechanism in BiVO_4_ as it is paired with WO_3_, representing an unintended consequence of composite formation. On the other hand, the photo-generated h^+^ at the VB of WO_3_ can transfer to the VB of BiVO_4_, which can oxidize H_2_O and HO^−^ to HO^•^. Adding to the complex combination of effects in the composite, the dye is seen to have a very strong affinity towards WO_3_. This is further explored by inspection of adsorption/desorption equilibrium of the dye, as measured in the dark (MB in Fig. 8 and RhB in Fig. S13), which shows the affinity of the dye towards WO_3_ to be quite strong as compared to BiVO_4_. In spite of an overall increased photocatalytic performance of the composite, as compared to pure WO_3_ or pure BiVO_4_, it can be seen that the mechanism of this enhancement is not straight forward. A similar trend was seen for RhB (see Fig. S13).

The role of surface area and isoelectric points (IEP) was explored (Fig. 8 above, as well as Fig. S14). This shows dye loading to be (1) highly dependent upon the materials’ isoelectric point and (2) for composites, loading is close to a stoichiometric mixture of the loading of the individual component materials. The effect of the affinity for the dye for the various metal oxides is pivotal towards our understanding of the photocatalytic behaviour of composites, as described in the following paragraphs.

Under illumination TiO_2_, can generate HO^•^ by reaction of adsorbed H_2_O and h^+^ in its VB. Additionally, adsorbed O_2_ can produce O_2_^•−^, by reaction with free e^−^ in the CB. Figure 7f shows the mechanisms observed in BiVO_4_/TiO_2_ composites, where the CB and VB of BiVO_4_ are both located between the CB and VB of TiO_2_. This represents an interesting case, particularly under 450 nm and 500 nm illumination, where only BiVO_4_ is photoexcited and there are no obvious pathways whereby CT to TiO_2_ can occur. Photocatalytic performance is however substantially higher than for pure BiVO_4_. This appears to again stem from the affinity of the dye for TiO_2_, whereby a high concentration of these dyes is maintained in proximity to the BiVO_4_ catalyst, which is the primary producer of the active radical species. This affinity is seen in the dark adsorption/desorption equilibrium (Fig. 8) and explained by the by the zeta potential of TiO_2_ (Fig. S14). Thus, the major process of this system should be driven by the visible light havering of BiVO_4_ which can produce HO_2_^•^ and/or O_2_^•−^ at its CB.

As CeO_2_ has a higher CB edge potential than BiVO_4_, free electrons have a substantial free energy to generate O_2_^•−^ from O_2_, which was seen to be the main photocatalytic mechanism at play here (Fig. 7d). The comparatively low photocatalytic activity of CeO_2_ (versus BiVO_4_) is in part attributed to low visible light absorption. 400 nm monochromatic illumination shows however that this is not the only factor, along with dark adsorption/desorption measurements (Fig. 8) which reveal the low affinity of the dye for CeO_2_, despite its high surface area. As seen in Fig. 7g, the CB and VB potential edges of BiVO_4_ are more positive than those of CeO_2_. The tendency of CT is that photoexcited e^−^ in the CB of CeO_2_ can be transferred to BiVO_4_, where they can react with absorbed O_2_ to create O_2_^•−^ radicals. From the quenching studies, HO^•^ was also observed to play role in this system, suggesting that these HO^•^ were probably generated from intermediate species and/or the generated O_2_^•−^ and HO_2_^•^ radicals^22, 24^. Therefore, the main mechanism is production of HO^•^ at the VB of the BiVO_4_ in the composite that leads to showing the lowest photocatalytic activity as compared to the CeO_2_/BiVO_4_ and TiO_2_/BiVO_4_ composites.

## Discussion

A series of composites were constructed, containing WO_3_, TiO_2_ or CeO_2_ with BiVO_4_. This last component ensured each system had similar overall light harvesting. XRD, XPS, SEM and TEM analyses confirm the formation of the composite without significant doping. Furthermore, highly porous films were formed, with a good dispersion of the different materials among each other.

Superior photocatalytic performance for degradation of MB and RhB under simulated solar light irradiation was observed for all composites, relative to their component materials. Monochromatic illumination and active species quenching studies were conducted and revealed a great deal of information about the different routes by which radical species were produced, leading to the degradation of these dyes. Quenching studies, indicated that O_2_^•−^ is a main reactive specie generated by CeO_2_/BiVO_4_ systems, while BiVO_4_/WO_3_ primarily degraded dyes via HO^•^, and both O_2_^•−^ and HO^•^ were active for the TiO_2_/BiVO_4_ composite.

Typically enhanced free carrier lifetimes resulting from a spatial separation of charges in different (neighbouring) materials is cited as a reason for enhanced performance, however here we see a number of other factors also had a bearing on the behaviour of composite systems. Specifically, it was seen that the affinity of the dye to the partner semiconductor plays a major role as it provides a high local concentration, where generated radicals have a higher probability of being able to react as compared to in the general solution. This was demonstrated clearly in the case the BiVO_4_-TiO_2_ composite, under 450 and 500 nm illumination of where no CT mechanisms are favourable, yet the degradation rate constant is roughly twice as large as for pure BiVO_4_. The WO_3_-BiVO_4_ composite was also seen to be an interesting case, with the band edge configuration deactivating one radical species generation pathway. Again, the overall performance increased, which again be attributable to high dye affinity for WO_3_.

This study highlights the complexities of composite design for photocatalysis, and the need to consider a range of factors, such as zeta potential and surface area, when designing new systems. It also demonstrated that band edge offsets can lead to deactivation of radical species generating pathways, as well as the more desirable increase in free charge carrier lifetime. We hope that it can serve as a basis for further design of new composite materials for water purification and other applications.

## Methods

### Preparation of photocatalyst powder

The synthesis of all photocatalysts were carried out by solution phase synthetic methods. For the synthesis of BiVO_4_ powder, bismuth nitrate and ammonium vanadate in dilute nitric acid solution were used as starting precursors for precipitation at room temperature. CeO_2_/BiVO_4_ and TiO_2_/BiVO_4_ composites were prepared by two-step methods^20, 43^. Firstly, BiVO_4_ powder was synthesised by a precipitation method. Subsequently the BiVO_4_ powder was added into either cerium nitrate or titanium isopropoxide solutions to prepare composites by precipitation or sol-gel methods, respectively. BiVO_4_/WO_3_ composites were also synthesised by a two-step precipitation method, where WO_3_ powder was first synthesised using tungsten nitrate as the starting material. The WO_3_ powder was added into a solution with of Bi and V precursors. The choice of synthetic order relates to pH regulation for each step and stability of the partner material under these conditions. Detailed descriptions of the synthesis of each of the CeO_2_/BiVO_4_, TiO_2_/BiVO_4_ and BiVO_4_/WO_3_ composites in the supporting information.

### Fabrication of photocatalyst films

All films for photocatalytic testing and electrochemical measurements were prepared by a doctor blading technique^29^. Briefly, 0.5 g of the photocatalyst powder was used, along with 75 µL Triton X-100, 25 µL acetic acid and 4 mL ethanol. The slurry was ground with a mortar and pestle for 10 min, during which ethanol was added in small aliquots to break up larger agglomerates. The paste was then sonicated for a further 30 min. Films were obtained by blading the slurry (~100 µL) on either glass slides (film size = 20 mm × 40 mm) for photocatalytic experiment or (~12.5 µL) on FTO glass working electrodes (film size = 10 mm × 10 mm) for Mott-Schottky experiment. This was carried out on a heated (50 °C) surface, using Scotch Tape (3 M, 50 μm) as a spacer. These films were then annealed in air at 450 °C for 2 h, to produce mechanically stable films. The thicknesses of the resulting films were 2.8 ± 0.1 µm, as measured with a stylus profilometer (VeecoDektak 150).

### Physical characterization of photocatalyst materials

X-Ray Diffractometry (JDX-3530, JEOL, Japan) was completed using Cu K_α_ radiation (λ = 1.546 nm). The detection range was 10° and 70° with the step size of 0.2° (2θ/s) in continuous scanning mode. Film morphologies were investigated by a Scanning Electron Microscope (SEM, JSM-7500FA, JEOL, Japan) with the accelerating voltage and emission current as 5.0 kV and 10 μA, respectively. The chemical composition and electron structure of the bare CeO_2_, BiVO_4_ and BiVO_4_/CeO_2_ composite were measured by X-ray photoelectron spectroscopy (XPS, PHOIBOS 100 hemispherical energy analyser from SPECS) using Al, K_α_ radiation (1486.6 eV) in fixed analyser transmission mode. The binding energies were calibrated with reference to C 1 s line at 284.6 eV for hydrocarbon contamination

Films were optically characterized using a Shimadzu 3600 UV-Vis-NIR spectrophotometer, with integrating sphere attachment. Transmission and reflection measurements were made in order to determine the absorption. Images were taken on an FEI Helios G3 CX using the STEM3 + detector at 30 kV. EDS data was acquires using an Oxford instruments X-max^N^ 150 mm^2^ SDD detector with acquisition and processing performed with the AZtec application suite. Samples were prepared by sonicating a dilute dispersion of the material in ethanol and dropping on a carbon coated copper grid.

### Photocatalytic testing

Photocatalytic activities of the synthesized photocatalyst powder and films were studied by the degradation of either Rhodamime B (~25 µM, RhB) or methylene blue (~50 µM, MB) solutions under simulated solar illumination (AM1.5 G, 1 sun equivalent, 100 mW cm^−2^). For monochromatic experiments, ±20 nm band pass filters were applied to the above light source.

Photocatalyst films were placed put in a reactor with the dye solution. Prior to irradiation, the films were left overnight in the dye solution under continuous magnetic stirring in order to attain an adsorption/desorption equilibrium. At irradiation time intervals of 30 min, the dye solution was collected and measured using an UV-vis spectrophotometer (Agilent 8453 photodiode array). After that, the collected solution was put back into the reactor, and the photodegradation reaction resumed (<3 min intermission).

An indirect chemical probe method was employed to investigate the mechanisms of dye degradation. Scavengers of various possible active species, including isopropanol (IPA, an HO^•^ quencher), benzoquinone (BQ, O_2_^•−^ quencher) and ethylenediamine tetra acetic acid (EDTA, a h^+^ scavenger), were added at 1 mM during the photoreaction^35–40^.

Each experiment was repeated in triplicate, with the average value shown along with one standard deviation as an error bar.

## Electronic supplementary material

Supplementary Information
