# Supplementary material for: Composite Photocatalysts Containing BiVO4 for Degradation of Cationic Dyes
Source: Sci Rep. 2017 Aug 21;7:8929. doi: 10.1038/s41598-017-09514-5 (PMC5567185; doi:10.1038/s41598-017-09514-5)
Supplement: Supplementary file 1 — Supplementary Information [file 41598_2017_9514_MOESM1_ESM.pdf]

Supplementary Information

Composite Photocatalysts Containing $BiVO_4$ for Degradation of Cationic Dyes

Kanlaya Pingmuang,[a,b] Jun Chen,[a] Wiyong Kangwansupamonkon,[c] Gordon G. Wallace,[a] Sukon Phanichphant,*[b] and Andrew Nattestad*[a]

[a] ARC Centre of Excellence for Electromaterials Science, Intelligent Polymer Research Institute, Australian Institute of Innovative materials, Innovation Campus, University of Wollongong, Fairy Meadow, NSW, 2519, Australia

[b] Department of Chemistry and Materials Science Research Center, Faculty of Science, Chiang Mai University, 239 Huay Kaew Road, Muang District, Chiang Mai, 50200, Thailand.

[c] National Nanotechnology Center, Thailand, 130 Thailand Science Park, Paholyothin Road, Pathumthani, 12120, Thailand.

Preparation of $BiVO_4$ powder

Pure $BiVO_4$ was synthesized by the homogeneous precipitation method using bismuth nitrate pentahydrate ($Bi(NO_3)_3{\cdot}5H_2O$, Sigma-Aldrich) and ammonium meta-vanadate (NH_4VO_3, Aldrich) as bismuth and vanadium precursors, respectively. In a typical procedure, 0.125 M each of Bi^{3+} and V^{5+} precursors were separately prepared by dissolving $Bi(NO_3)_3{\cdot}5H_2O$ in 3 M nitric acid (HNO_3) solution and NH_4VO_3 in 3 M

ammonium hydroxide (NH_4OH) solution. The vanadium precursor solution was slowly added to the bismuth nitrate solution under stirring over 30 min. Then 3 M ammonia solution was added drop wise until pH 7 was attained. The resultant precipitate was washed with deionized water, centrifuged and dried at 60°C for 12 h. Finally, the dried powder was then calcined at 450°C for 2 h to obtain $BiVO_4$ powder.

Preparation of CeO_2 and $CeO_2/BiVO_4$ composite powder

Pure CeO_2 and $BiVO_4/CeO_2$ nanocomposite catalysts were prepared with different mole ratios (4:1, 3:2, 1:1, 2:3 and 1:4.) by a precipitation process. Briefly, Cerium(III)nitrate hexahydrate ($Ce(NO_3)_3{\cdot}6H_2O$, Sigma-Aldrich) was dissolved in 100 mL of 80% v/v ethylene glycol solution. The solution was kept under constant stirring and heated at 50°C until a homogeneous solution was obtained. The $BiVO_4$ powder was subsequently added to the above cerium precursor solution to prepare $BiVO_4/CeO_2$ composite with the desired mole ratio. After that, 25 mL of 3 M ammonium hydroxide (NH_4OH) was slowly added into the above solution, and the transparent solution changed to a yellowish suspension. The suspension was kept under stirring at 50°C for a further 24 h and the precipitate was finally collected by centrifugation, washed 3 times with deionized water and then dried at 60°C for 24 h. The obtained powder was subsequently calcined at 450°C for 2 h. The as-synthesized composites were characterized and actual the ratio of $BiVO_4$:CeO_2 was confirmed by elemental analysis of Bi (from to $BiVO_4$) and Ce (CeO_2). For control experiments, pure CeO_2 was also prepared by the procedure described above without the $BiVO_4$ addition step, which this synthetic method have been previously reported by our group.[27]

Preparation of TiO_2 and $TiO_2/BiVO_4$ composite powder

$BiVO_4/TiO_2$ nanocomposite catalysts with different mole ratios (4:1, 3:2, 1:1, 2:3 and 1:4.) were synthesized by coupling a precipitation and sol-gel methods. Firstly, pure $BiVO_4$ powder was synthesized as mention above. A sol-gel method of anatase TiO_2 synthesis, reported by Wetchakun *et al.* ,[28] was used here. Firstly, 20 mL titanium tetraisopropoxide (TTIP, Sigma-Aldrich) was dissolved in 250 mL 6 M nitric acid solution and mixed until a homogeneous solution was obtained. The mixture of TTIP and nitric acid solution was put into

a cellophane membrane and then placed in solution containing a 1:1 v/v ratio (350 mL) of absolute ethanol and deionized water with 0.5–1.0 vol% concentrated (25%) ammonia. The $BiVO_4$ powder was subsequently added to the above mixture in the cellophane pouch to synthesize $BiVO_4/TiO_2$ composite powders. The mixture inside the cellophane pouch was kept stirring with magnetic stirrer bar and heated to 80°C for 1 h. After the completion of the dialysis process, the suspension was centrifuged at 5000 rpm for 10 min, washed with deionized water and then dried in an oven at 60°C for 24 h. The obtained powder was finally calcined in a furnace at the temperature of 450°C for 2 h. For control experiments, pure TiO_2 photocatalyst was also synthesized by the procedure described above, without any $BiVO_4$ added.

Preparation of WO_3 and $BiVO_4/WO_3$ composite powder

$BiVO_4/WO_3$ nanocomposite photocatalysts with different mole ratios between were synthesized by a two-step precipitation processes, because the $BiVO_4$ powder is not stable in acidic conditions, WO_3 powder was first synthesized by dissolving 5 g of sodium tungstate dihydrate ($Na_2WO_4{\cdot}2H_2O$, Sigma-Aldrich) in a solution containing 10 g of citric acid, 75 mL of ethylene glycol and 25 mL of deionized water under continuous stirring. The solution was heated at 70°C, and then 25 mL of 1M hydrochloric acid (HCl) was added to the above solution (pH ~1). The mixed solution was kept under continuous stirring and maintaining a constant temperature until the formation of a yellow-green precipitate. This powder was separated by centrifugation, washed with deionized water and ethanol several times, dried at 60°C for 24 h, and then calcined at 450°C for 2 h. This WO_3 powder was mixed into precursor solution used in $BiVO_4$ synthesis by precipitation.

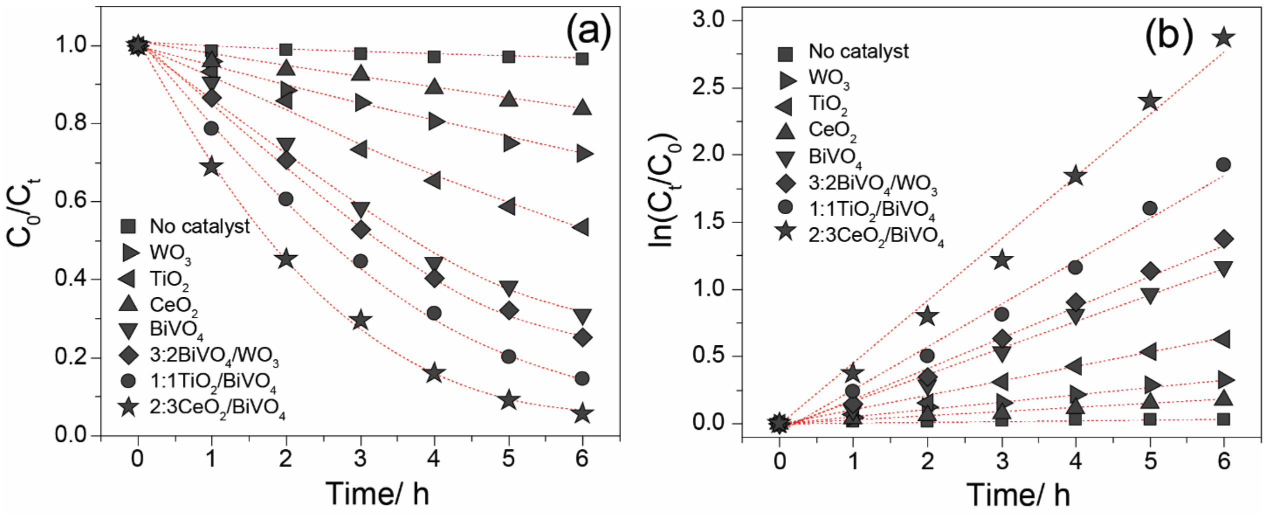

Figure S1 Comparison of (a) photocatalytic efficiencies and (b) their kinetic plots ($\ln(C_0/C_t)$ *vs. t*, $C_{ads/des} = C_0$) for degradation of RhB using different photocatalyst films under solar light irradiation.

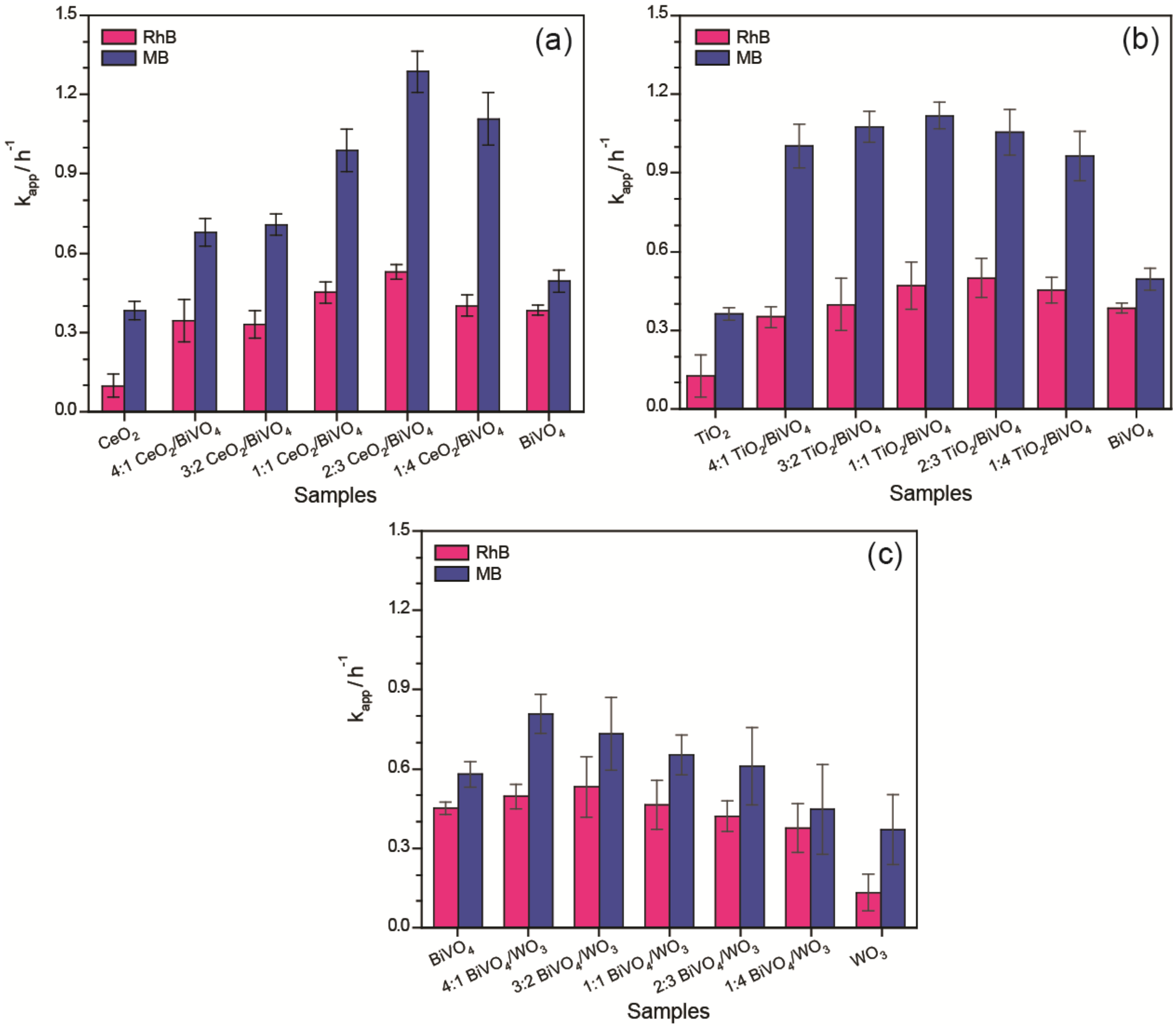

Figure S2 Pseudo first order rate constants of photodegradation of RhB and MB dyes by using (a) $CeO_2/BiVO_4$, (b) $TiO_2/BiVO_4$ and $BiVO_4/WO_3$ composite films with different mole ratio under irradiation of solar light irradiation.

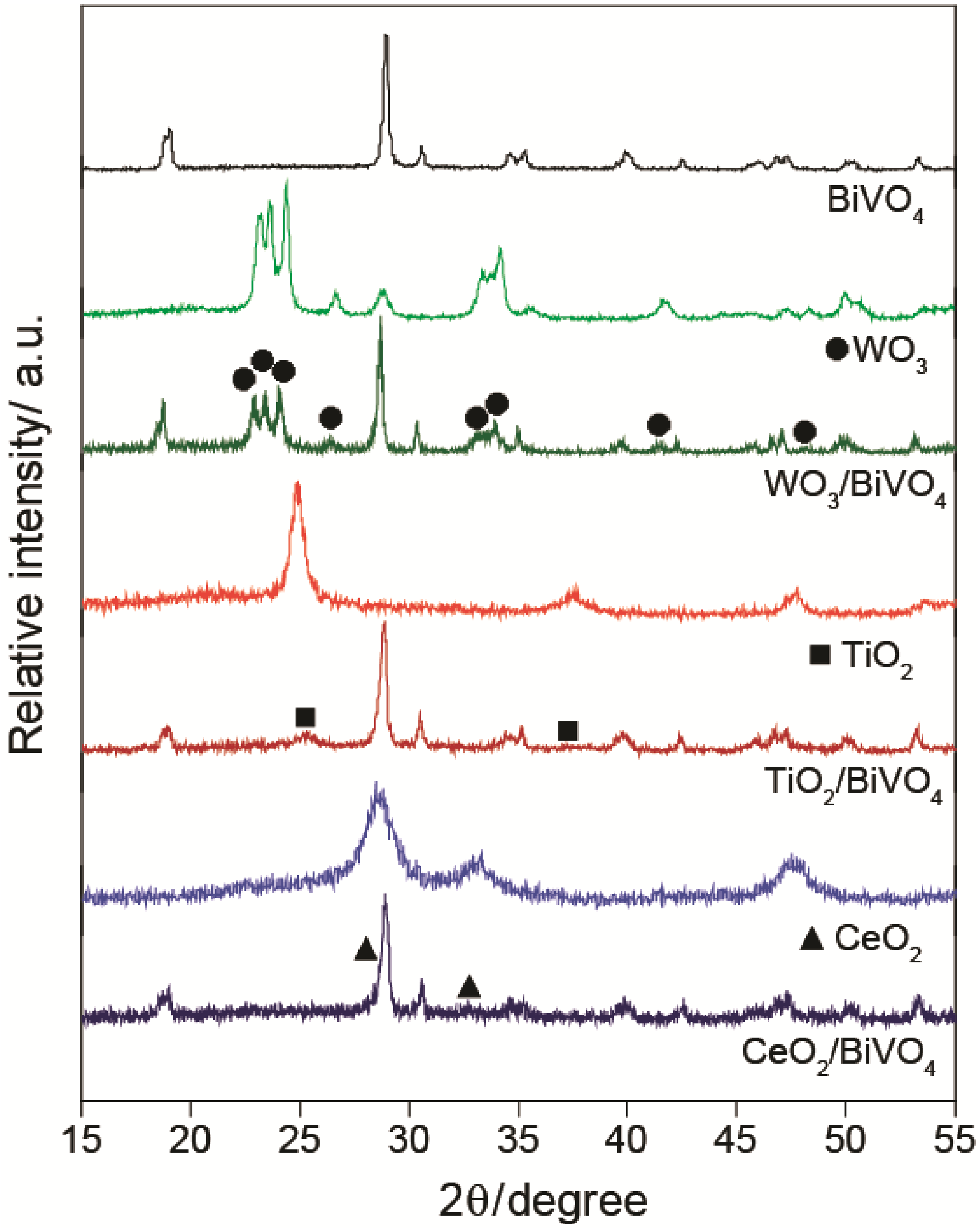

Figure S3 XRD patterns of composite and pure photocatalysts

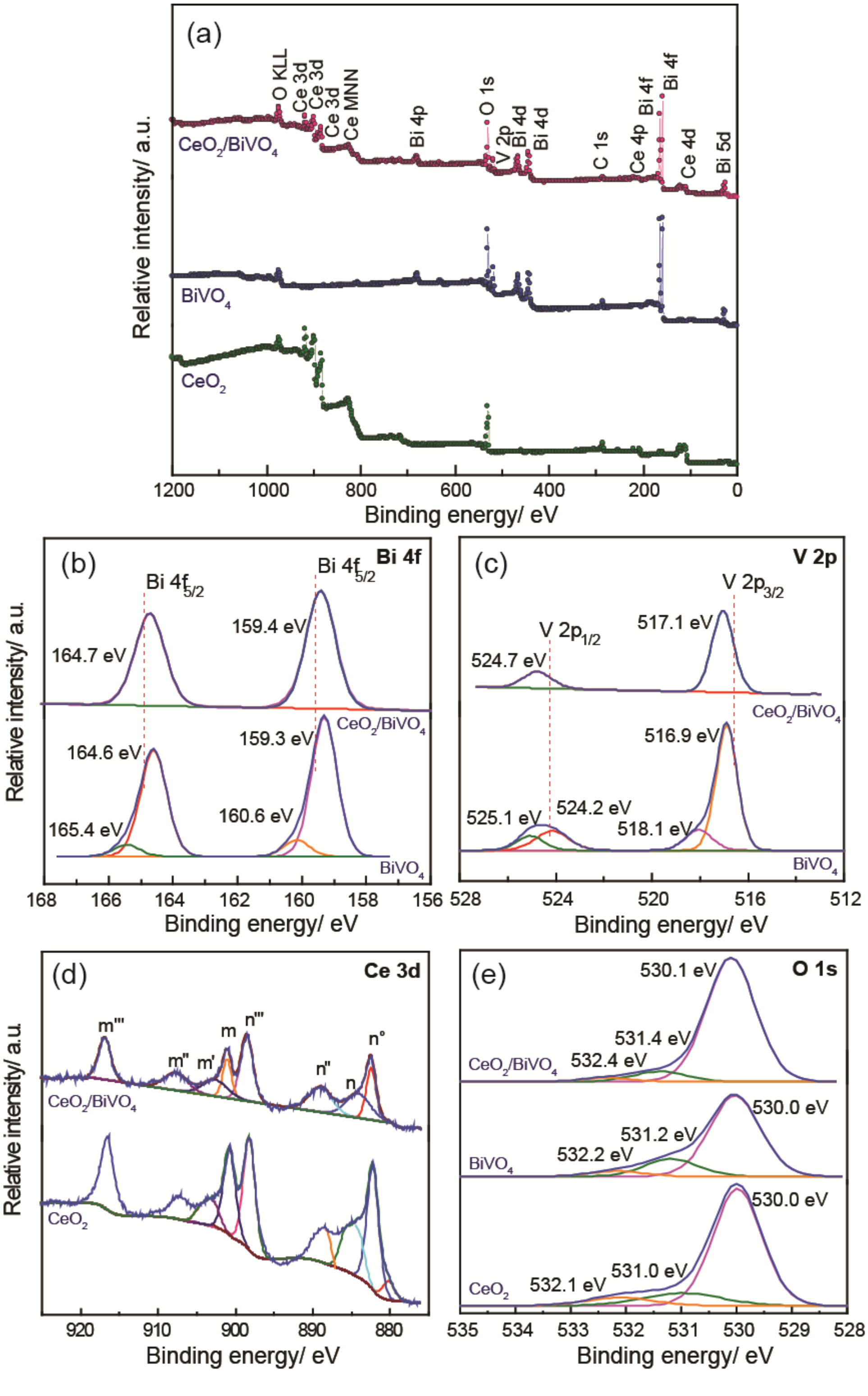

Figure S4 XPS survey spectra of $BiVO_4$, CeO_2 and 2:3 $CeO_2/BiVO_4$ composite, high-resolution XPS spectrum of (b) Bi 4f, (c) V 2p, (d) Ce 3d and (e) O 1s regions.

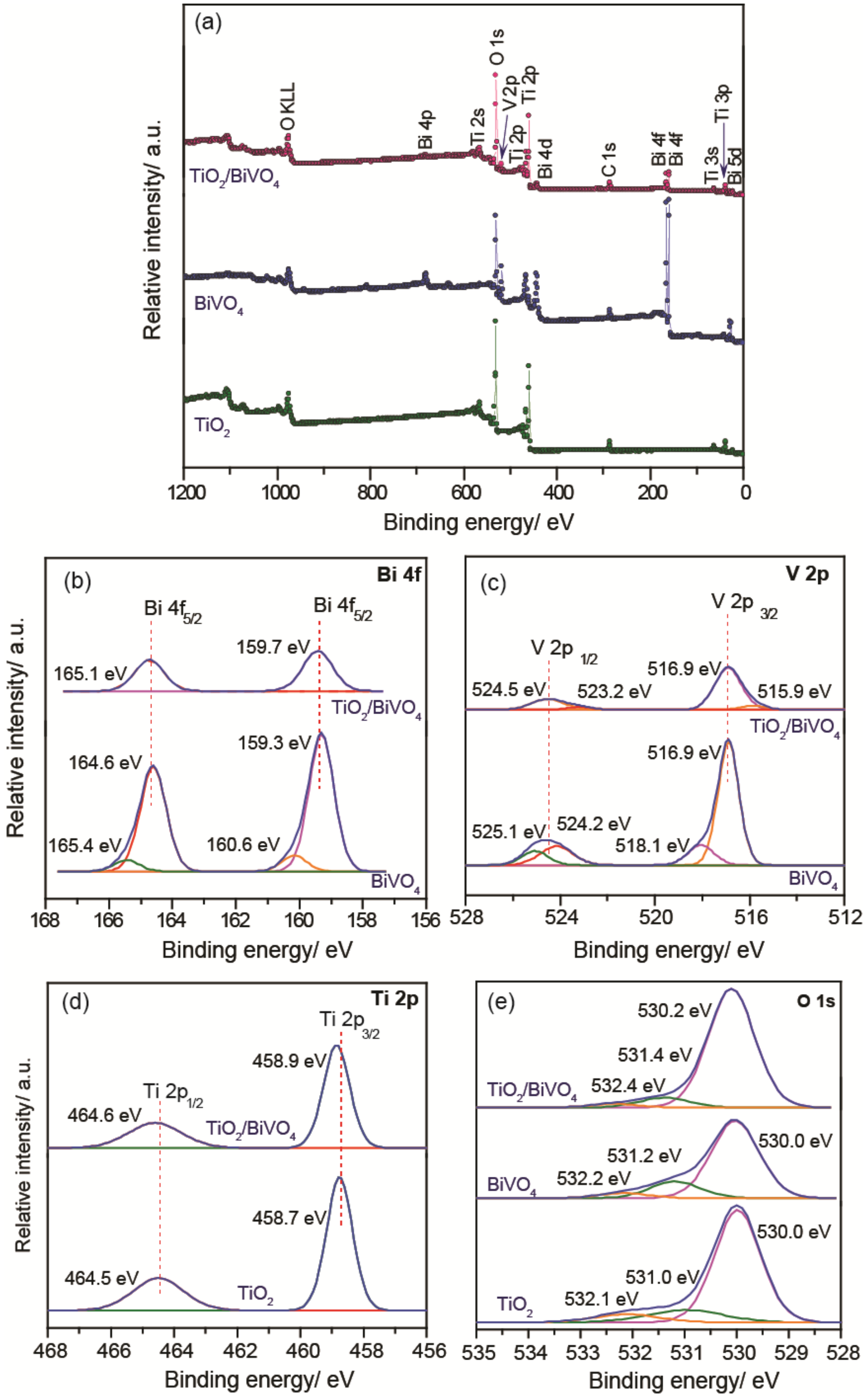

Figure S5 XPS survey spectra of $BiVO_4$, TiO_2 and 1:1 $TiO_2/BiVO_4$ composite, high-resolution XPS spectrum of (b) Bi 4f, (c) V 2p, (d) Ti 2p and (e) O 1s regions.

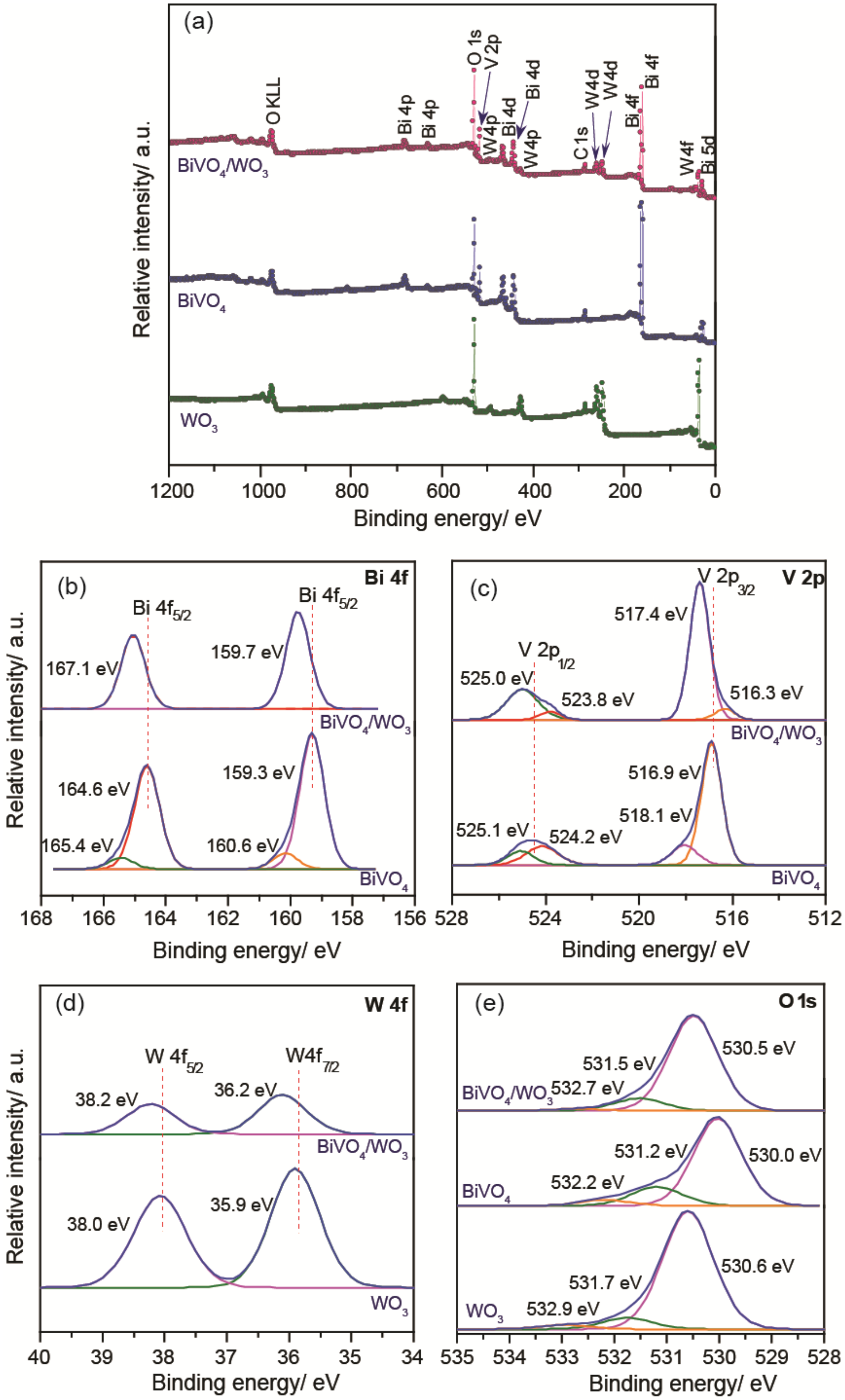

Figure S6 XPS survey spectra of $BiVO_4$, WO_3 and 4:1 $BiVO_4/WO_3$ composite, high resolution XPS spectrum of (b) Bi 4f, (c) V 2p, (d) W 4f and (e) O 1s regions.

Table S1 Crystalline size and surface area of composite photocatalysts

Materials	Crystalline size (nm)		S_{BET} (m^2 g^{-1})
	$BiVO_4$	Another metal oxide	
$BiVO_4$	20	-	13.6
CeO_2	-	-	147
$CeO_2/BiVO_4$	-	-	62.7
TiO_2	-	10	103
$TiO_2/BiVO_4$	17	8	49.9
WO_3	-	18	36.5
$WO_3/BiVO_4$	32	20	28.6

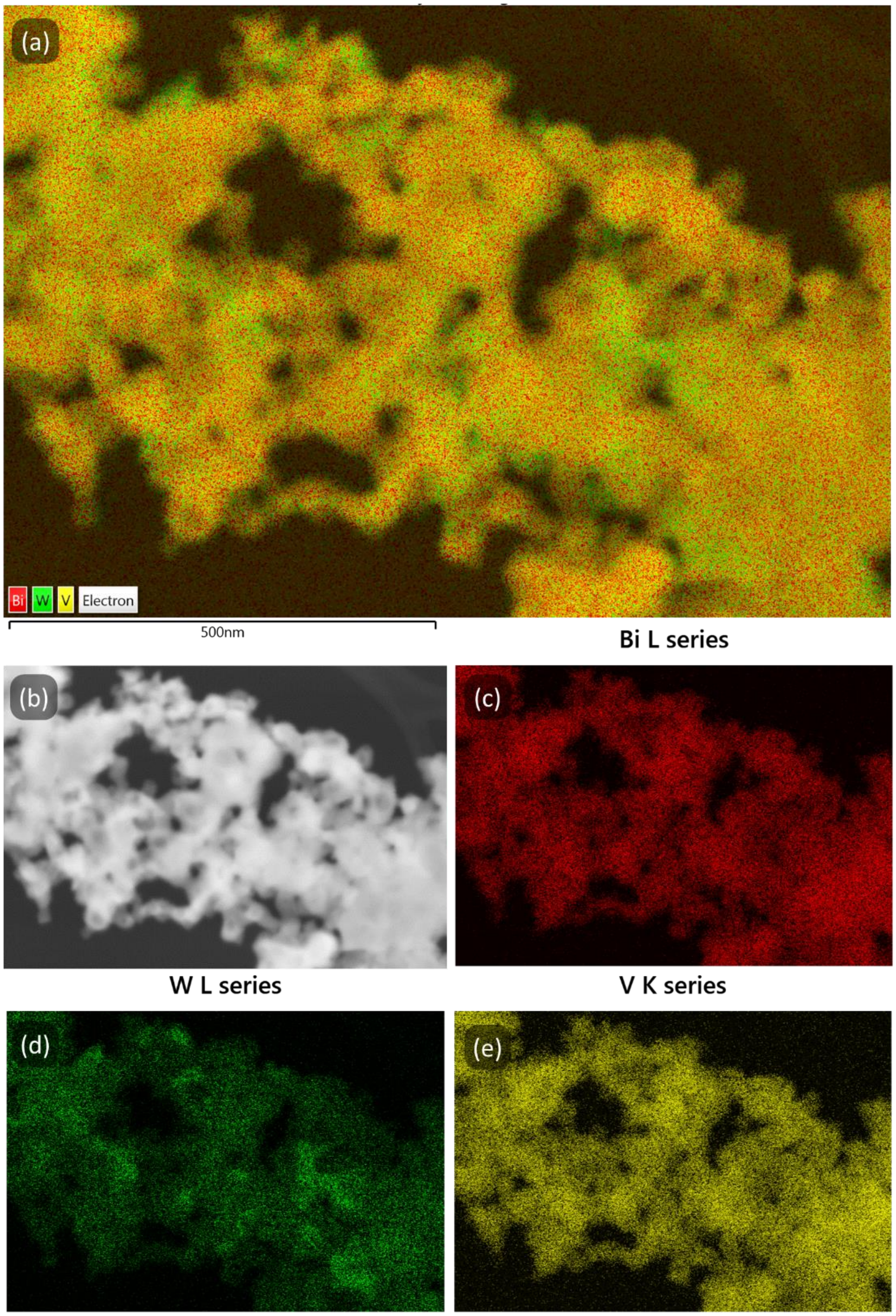

Figure S7 EDS maps of $BiVO_4/WO_3$, (a) composite map (b) SEM image elemental maps of (c) Bi (d) W and (e) V.

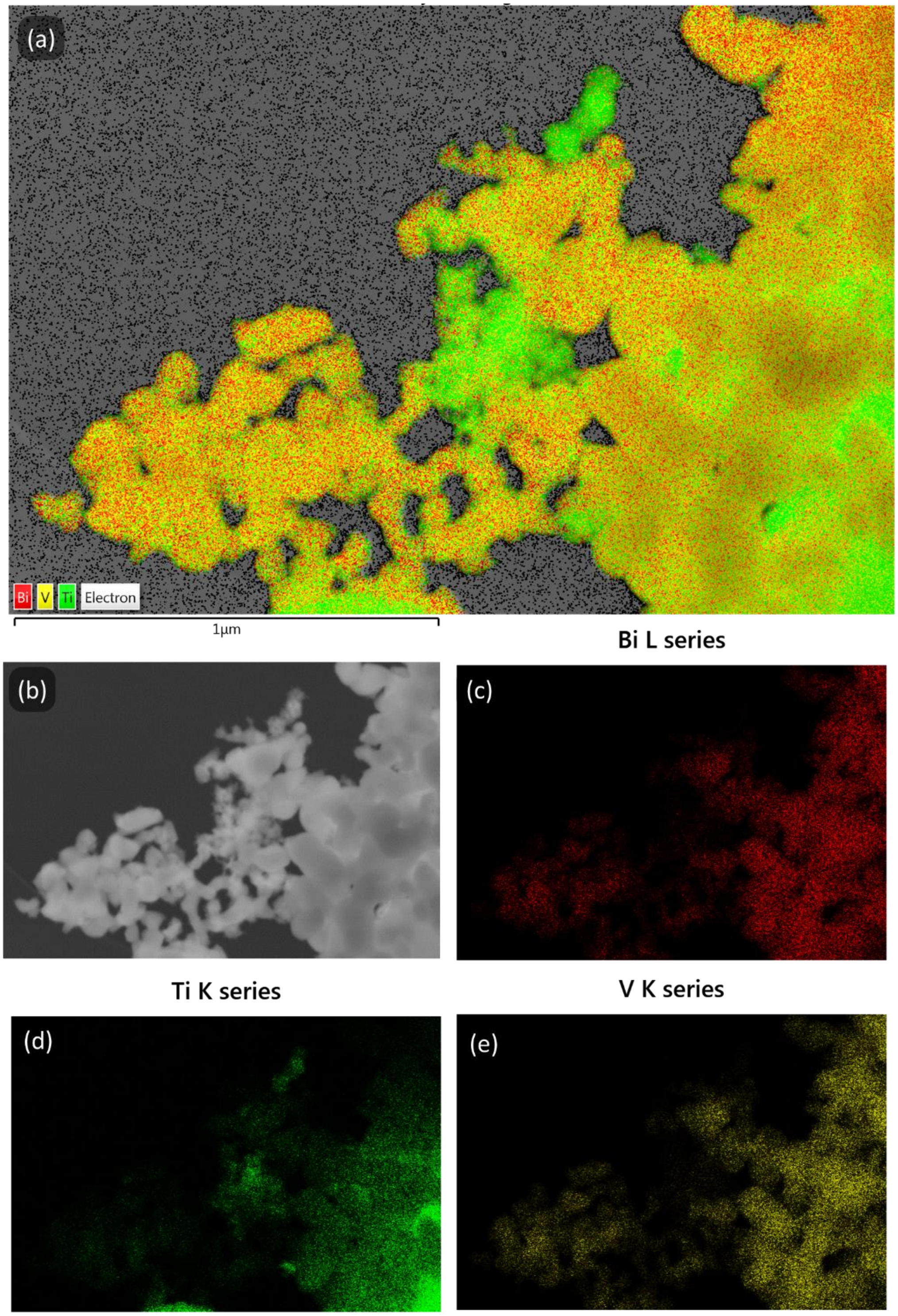

Figure S8 EDS maps of $BiVO_4/TiO_2$, (a) composite map (b) SEM image elemental maps of (c) Bi (d) Ti and (e) V.

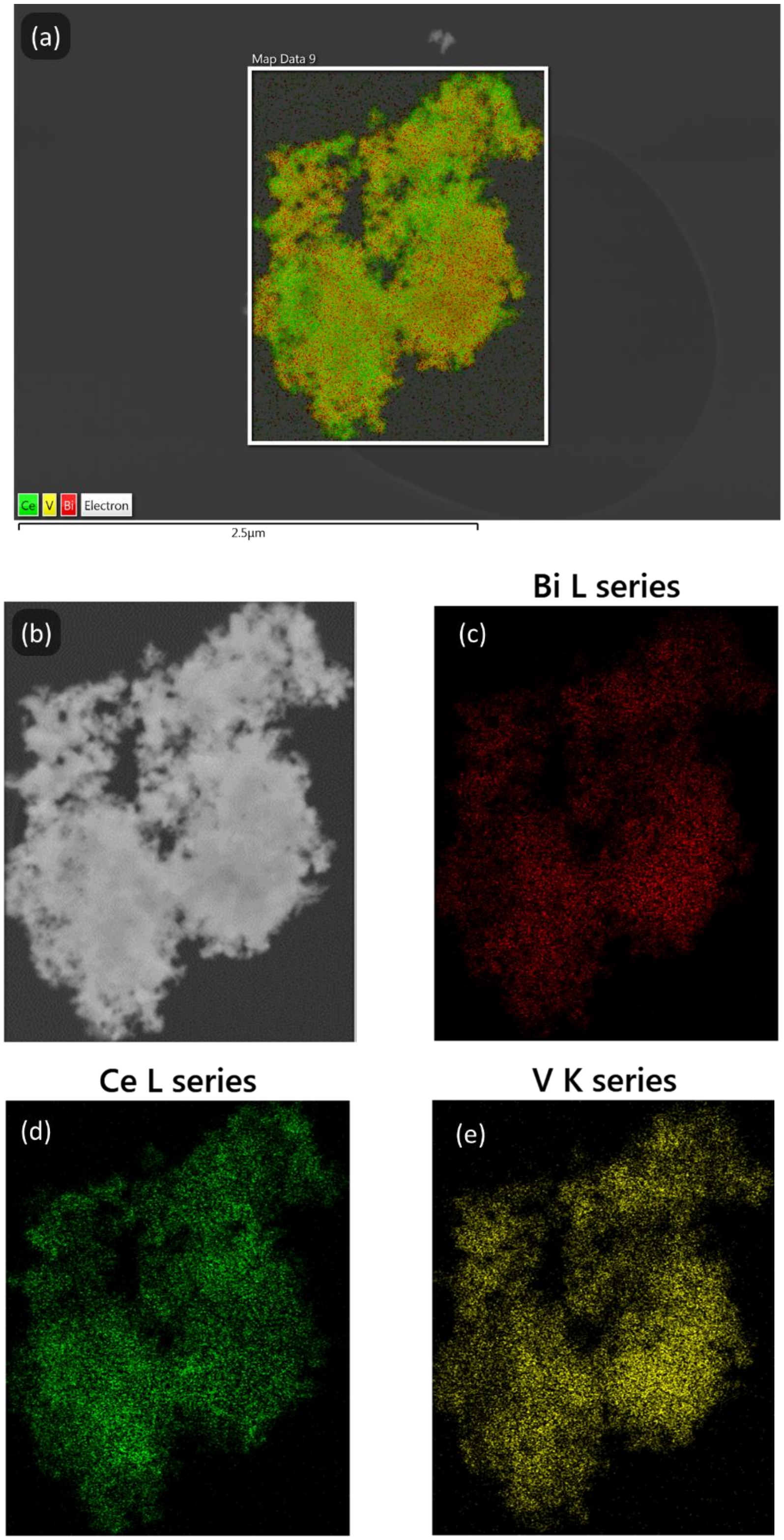

Figure S9 EDS maps of $BiVO_4/CeO_2$, (a) composite map (b) SEM image elemental maps of (c) Bi (d) Ce and (e) V.

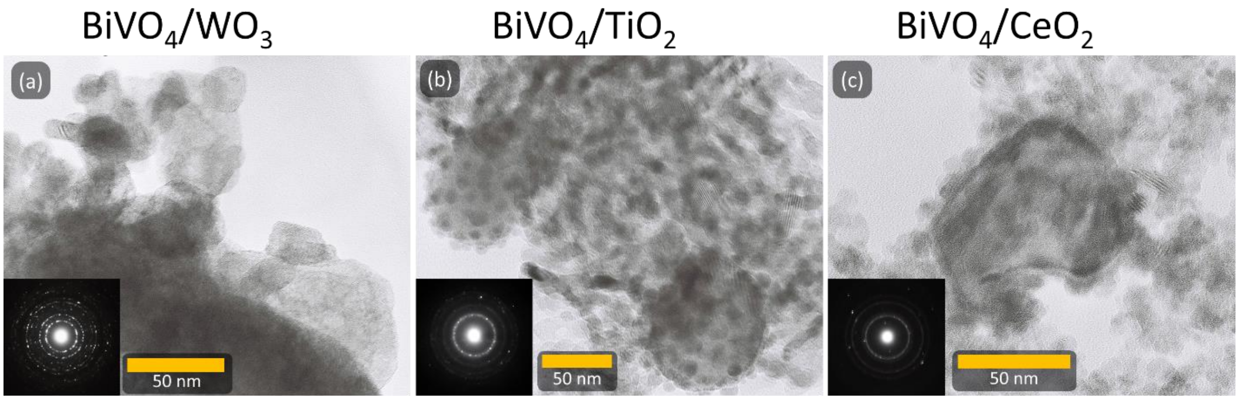

Figure S10 TEM images of composite photocatalyst powders containing (a) $BiVO_4$ and WO_3, (b) $BiVO_4$ and TiO_2 and (c) $BiVO_4$ and CeO_2.

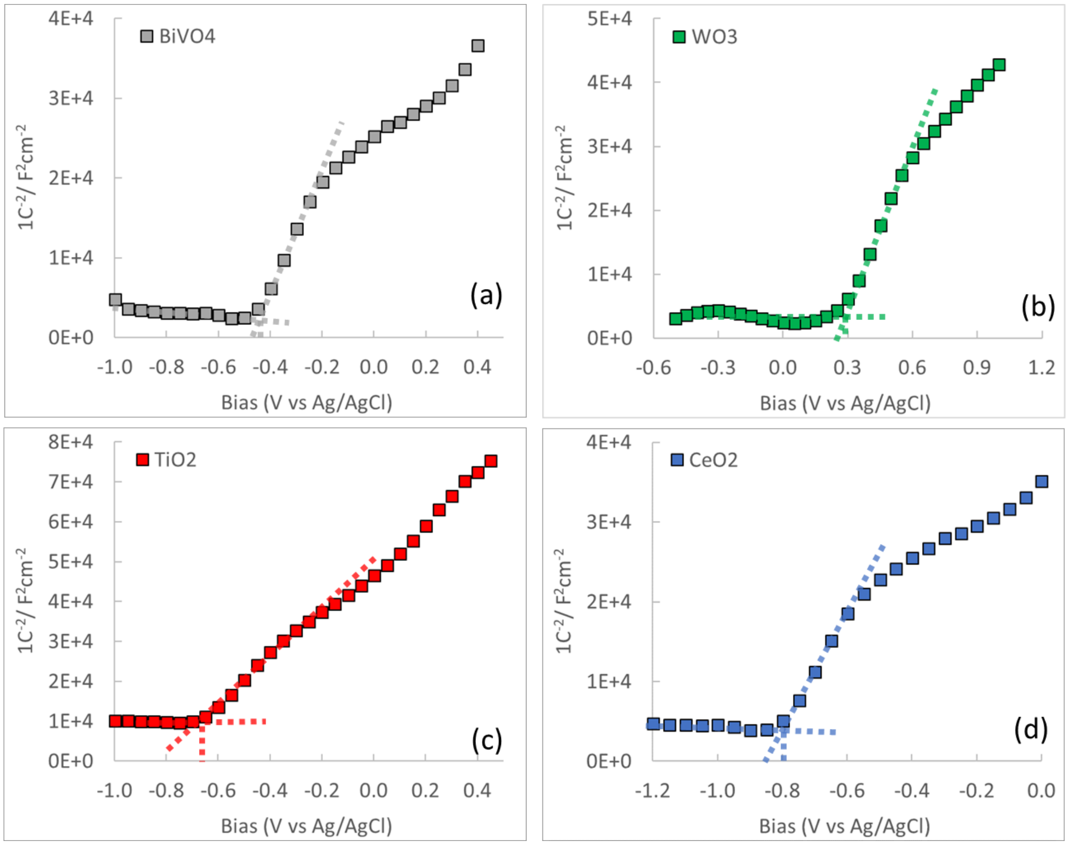

Figure S11 Mott-Schottky plots of (a) BiVO4, (b) CeO2, (c) TiO2 and (d) WO3 films on FTO substrates were carried out in 0.5 M Na2SO4 solution (pH 5.5) at frequency 1 kHz.

The measured potentials *vs.* the Ag/AgCl were converted to the reversible hydrogen electrode (RHE) scale via the Nernst equation:[3, 4]

$E_{RHE} = E_{Ag/AgCl} + E^0_{Ag/AgCl\ vs.\ NHE} + 0.059 \times pH$

At pH = 5, $E_{RHE} = E_{Ag/AgCl} + 209\ mV + 295\ mV = E_{Ag/AgCl} + 504\ mV$

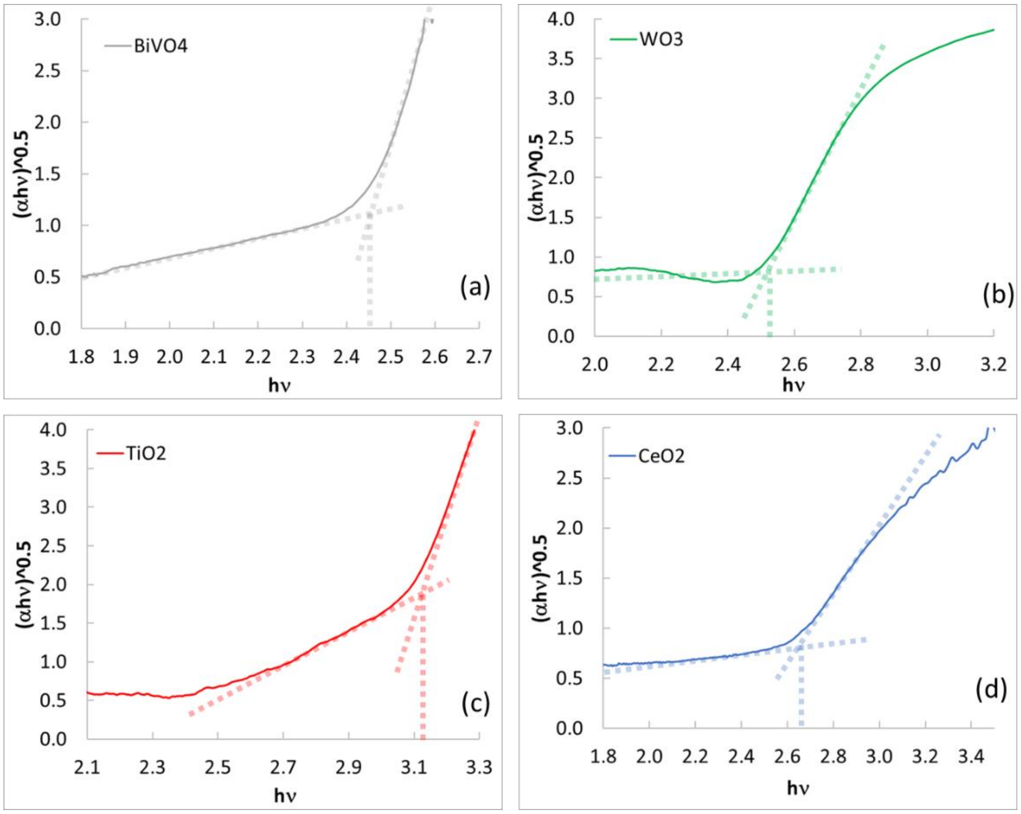

Figure S12 Kubelka Munk transformation of absorption spectra of (a) BiVO4, (b) CeO2, (c) TiO2 and (d) WO3 films on FTO substrates were carried out in 0.5 M Na2SO4 solution (pH 5.5) at frequency 1 kHz.

Table S2 Summary of the calculated band edge energies of individual semiconductors from Mott-Schottky results.

Materials	E_{fb} *vs.* Ag/AgCl	CB (E *vs.* RHE)	E_g (eV)	VB (E *vs.* RHE)
$BiVO_4$	-0.43	0.09	2.45	2.54
CeO_2	-0.8	-0.28	2.65	2.37
TiO_2	-0.68	-0.37	3.15	2.78
WO_3	0.31	0.83	2.55	3.38

Table S3 The wavelength of the monochromatic light and the corresponding power used in the photocatalytic tests under the monochromatic light.

**Wavelength (nm)**	400 (382–424)	450 (436–478)	500 (484–522)	550 (536–577)	600 (586–623)	650 (640–676)	700 (684–723)
**Filtered Si diode response (μA)**	10	49	59	67	49	23	11
**% EQE**	46.3	59.7	67.8	68.1	52.6	25.5	8.05
**relative flux**	0.215	0.820	0.871	0.984	0.931	0.901	1.37

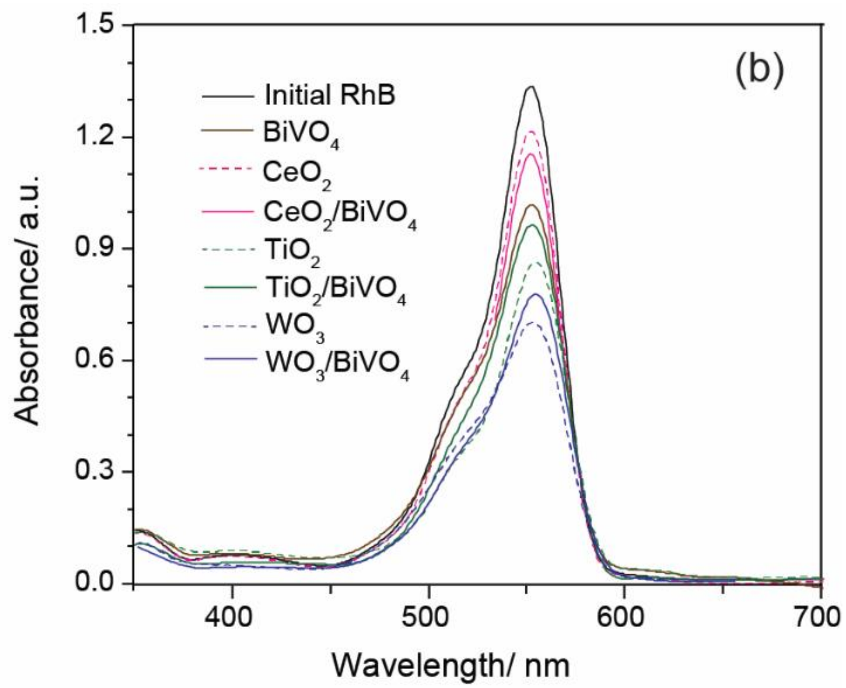

Figure S13 Absorption spectra of RhB after 24h in the dark where an adsorption/desorption equilibrium is obtained.

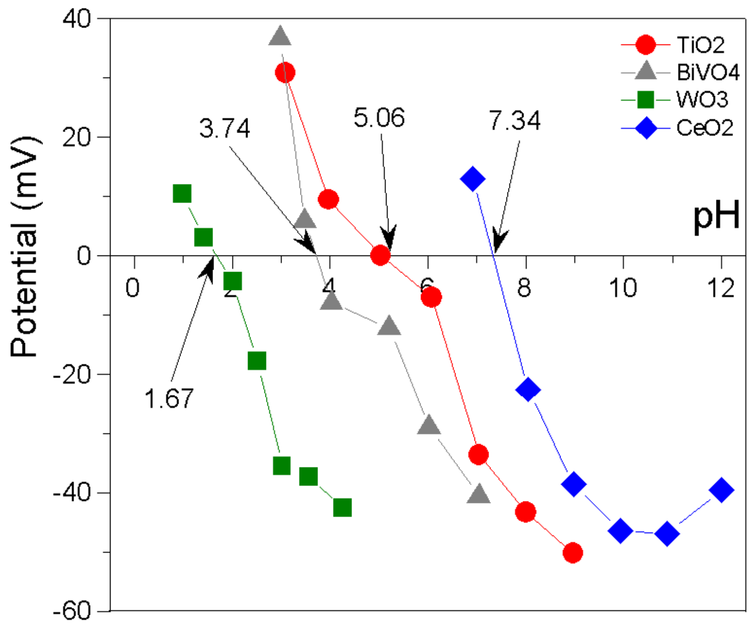

Figure S14 pH dependence of zeta potentials of $BiVO_4$, CeO_2, TiO_2 and WO_3 in aqueous solutions.

References

1. N. Wetchakun, S. Chaiwichain, B. Inceesungvorn, K. Pingmuang, S. Phanichphant, A. I. Minett and J. Chen, *ACS Appl. Mater. Interfaces*, 2012, **4**, 3718–3723.

2. N. Wetchakun, B. Incessungvorn, K. Wetchakun, S. Phanichphant, *Mater. Lett.*, 2012, **82**, 195–198.

3. F. F. Abdi and R. V. D. Krol, *J. Phys. Chem. C*, 2012, **116**, 9398–9404.

4. S. Hoang, S. Guo, N. T. Hahn, A. J. Bard, and C. B. Mullins, *Nano Lett.*, 2012, **12**, 26–32.
